# Psychometric properties and population norms of the Brief Symptom Inventory-18 (BSI-18) based on a representative German sample

**DOI:** 10.3389/fpsyt.2026.1877721

**Published:** 2026-08-12

**Authors:** Sören Kliem, Anna Lohmann, Sebastian Fischer, Markus Zenger, Dirk Baier, Elmar Brähler, Jörg M. Fegert, Nora Hettich-Damm, Cedric Sachser

**Affiliations:** 1 Department of Social Welfare, Ernst-Abbe University of Applied Sciences Jena, Jena, Germany; 2 Faculty of Applied Human Studies, University of Applied Sciences Magdeburg and Stendal, Stendal, Germany; 3 Integrated Research and Treatment Center Adiposity Diseases - Behavioral Medicine Psychosomatic Medicine and Psychotherapy, University of Leipzig Medical Center, Leipzig, Germany; 4 Institute of Delinquency and Crime Prevention, Zurich University of Applied Sciences, Zurich, Switzerland; 5 Crimelab, University of Zurich, Zurich, Switzerland; 6 Department of Psychosomatic Medicine and Psychotherapy, University Medical Center of the Johannes Gutenberg University Mainz, Mainz, Germany; 7 Clinic and Polyclinic for Psychosomatic Medicine and Psychotherapy, Rostock University Medical Center, Rostock, Germany; 8 Department of Child and Adolescent Psychiatry and Psychotherapy, Ulm University Medical Center, Ulm, Germany

**Keywords:** anxiety, BSI-18, depression, measurement invariance, population norms, psychological distress, psychometric properties, somatization

## Abstract

**Background:**

The Brief Symptom Inventory-18 (BSI-18) is a widely used self-report measure of psychological distress assessing somatization, depression, and anxiety. Although German population norms were published in 2017 based on data from 2009, updated psychometric evidence using modern statistical methods is lacking.

**Methods:**

The BSI-18 was administered to a random probability sample representative of the German residential population aged 16 years and older (N = 2,519; 52.5% women; age range 16–96 years), collected between December 2020 and March 2021. Psychometric evaluation included confirmatory factor analysis (CFA) with the weighted least squares mean and variance adjusted (WLSMV) estimator, internal consistency (McDonald’s omega and Cronbach’s alpha with bootstrap confidence intervals), measurement invariance across gender, age, and clinical screening status, construct validity via latent correlations with the PHQ-9, GAD-7, and CFQ-11, and age- and gender-stratified population norms. Cross-cohort comparisons were conducted with the normative sample from 2009.

**Results:**

The correlated three-factor model showed good fit (χ^2^(132) = 644, CFI = 0.986, RMSEA = 0.039, 90% CI [0.036, 0.042]). Internal consistency was good to very good (Global Severity Index [GSI]: ω = 0.966; subscales: ω = 0.855 to 0.893). Full measurement invariance was established across gender, age, and clinical screening status. Latent correlations confirmed the hypothesized convergent validity pattern for depression (PHQ-9: *r* = 0.912), anxiety (GAD-7: *r* = 0.889) and somatization (CFQ-11: *r* = 0.580). Women scored higher on all scales (*d* = -0.11 to -0.14). Cross-cohort comparisons revealed no significant change in the GSI (*d* = -0.05, *p* = 0.105) or depression (*d* = 0.04, *p* = 0.200), whereas somatization (*d* = -0.11, *p* < 0.001) and anxiety (*d* = -0.07, *p* = 0.006) scores were significantly lower in 2020/21.

**Conclusions:**

The German BSI-18 demonstrated sound psychometric properties, including full measurement invariance. The updated population norms provide a useful reference for clinical practice. Cross-cohort comparisons revealed no general increase in distress during the COVID-19 pandemic.

## 1 Introduction

Somatization, depression, and anxiety represent the three most prevalent dimensions of psychological distress in the general population as well as in primary care settings (1). Epidemiological data consistently indicate that these conditions are highly comorbid and associated with substantial individual suffering, functional impairment, and societal costs. Early identification of psychological distress is therefore a priority in both clinical and public health contexts, and efficient screening instruments play a key role in this endeavor.

Self-report measures of psychological distress have a long tradition in clinical psychology and psychiatry. Among the most established instruments internationally is the family of measures originating from the Hopkins Symptom Checklist [HSCL (2)], which was systematically extended and revised into the 90-item Symptom Checklist-90-Revised [SCL-90-R (3, 4), German version (5):]. While the SCL-90-R has been translated into many languages (6) its length limits applicability in time-sensitive settings. To address this limitation, Derogatis and Melisaratos (7) developed the 53-item Brief Symptom Inventory [BSI (8), German version (9):], retaining the nine-dimensional structure. Subsequently, Derogatis (10) introduced the BSI-18 as an even shorter version, focusing on the three clinically most relevant dimensions: somatization, depression, and anxiety.

The BSI-18 (10) comprises 18 items assessed on a five-point Likert scale from 0 (not at all) to 4 (extremely), with reference to the past seven days. It yields three subscale sum scores for somatization (SOM; items 1, 4, 7, 10, 13, 16), depression (DEP; items 2, 5, 8, 11, 14, 17), and anxiety (ANX; items 3, 6, 9, 12, 15, 18), each ranging from 0 to 24, as well as a Global Severity Index (GSI; range 0-72) computed as the sum of all 18 items. Since its publication, the BSI-18 has been widely adopted in psycho-oncology (11, 12), geriatric populations (13), forensic and addiction medicine (14), and epidemiological studies (15).

The theoretical three-factor structure has been supported by confirmatory factor analyses (CFA) in a variety of populations, including childhood cancer survivors (16), drug users (14), Spanish cancer patients (17, 18), and in a meta-analytic CFA (19). However, findings are not entirely consistent. Some studies have reported better fit for a four-factor solution separating panic from general anxiety (20, 21) and several studies have found that a bifactor model provided better fit (22–25). Using item response theory, Meijer et al. (26) identified substantial variability in item discrimination, with item 17 (thoughts of ending your life) being the least informative for the underlying distress continuum. Measurement invariance has been tested across gender (17, 22, 27), ethnicity (14, 28, 29), and nativity and language format (27). To date, however, measurement invariance has not been examined in a representative German sample.

The availability of current population norms is essential for the clinical interpretation of screening instruments. Symptom levels in the general population are not static but change over time due to demographic shifts, changing health behaviors, and societal stressors (30–32). Updated normative data are essential to ensure that clinicians can interpret scores within a contemporary reference frame (33). The only existing German norms for the BSI-18 were published by Franke et al. (34) based on data collected in 2009. However, their psychometric analysis employed the asymptotically distribution-free (ADF) estimator, yielding implausibly poor model fit (CFI = 0.55), and their norms may no longer reflect the current symptom burden in the population. Although the present data were collected during the COVID-19 pandemic (December 2020 to March 2021), they constitute the most recent representative assessment of the BSI-18 in Germany. The concurrent comparison with the 2009 normative sample provides an empirical test of whether symptom levels during this period deviated from the pre-pandemic reference, and thus of whether the updated norms can serve as a valid reference beyond the pandemic period.

The aims of the present study were: (i) to provide updated population norms for the BSI-18 based on a new representative German sample, (ii) to evaluate factorial validity using the weighted least squares mean and variance adjusted (WLSMV) estimator, (iii) to assess internal consistency using McDonald’s omega and Cronbach’s alpha with bootstrap confidence intervals, (iv) to test measurement invariance across gender, age, and clinical screening status, (v) to evaluate construct validity through latent correlations with measures of depression (PHQ-9), anxiety (GAD-7), and fatigue (CFQ-11), and (vi) to conduct cross-cohort comparisons with the 2009 normative sample.

## 2 Methods

### 2.1 Procedure

Data were collected between December 2020 and March 2021 as part of a representative German community survey conducted by Leipzig University. The goals of the survey were (a) to assess prevalence rates of relevant physical or mental disorders and related risk behaviors, (b) to examine causes and conditions of these disorders, and (c) to analyze psychometric properties and provide German population norms for clinical-psychological instruments. Data collection was carried out by a demographic research institute (USUMA GmbH, Berlin), an independent social research institute specializing in population-based survey research. The study was approved by the Ethics Committee of the Medical Faculty of the University of Leipzig (Az.: 474/20-ek). All participants provided written informed consent. For under-aged participants, at least one legal guardian was informed about the sampling procedure and survey contents. The study followed the Declaration of Helsinki.

The sampling procedure followed the ADM (Arbeitskreis Deutscher Markt- und Sozialforschungsinstitute) face-to-face design. In a first step, *k* = 258 sample points were selected using Cox allocation proportional to the population of each federal state. In a second step, *n* = 5,934 target households were selected using a random-route procedure. In a third step, the target person within each household was identified using the Kish method (35). Representativeness was thus ensured by design: sample points were allocated proportionally to the population across all federal states, and households and target persons were selected at random without quota elements. No post-stratification weighting was applied. The minimum age for participation was 16 years. After removing 21 quality-neutral cases (e.g., uninhabited dwellings, no target person in household), the net sample was 5,913. Of these, 2,556 interviews were completed (response rate: 43.1%), of which 2,519 were evaluable after quality checks. Data collection comprised two parts: (a) an interviewer-administered section covering sociodemographic characteristics following the standards of the German Federal Statistical Office (Statistisches Bundesamt), and (b) a paper-based self-report questionnaire containing the psychological instruments. Interviewers remained available for questions but did not interfere with responses.

### 2.2 Sample

**Figure 1:**
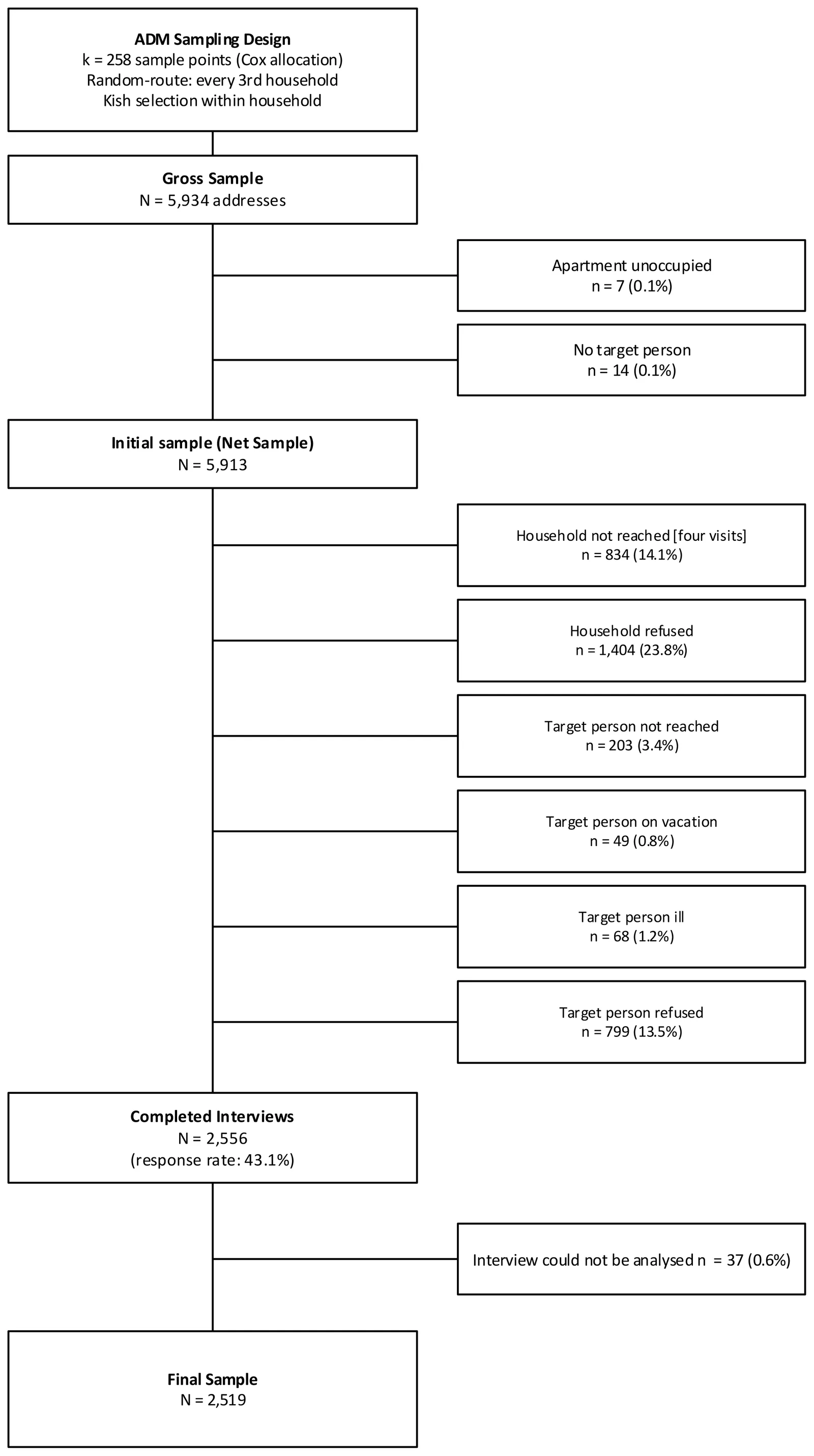
Participant flowchart and data preparation procedure.

Data from *N* = 2,519 participants (mean age: 50.3 years, *SD* = 18.1, range: 16-96) were analyzed. The sample comprised *n* = 1,193 men (mean age: 50.1, *SD* = 17.7), *n* = 1,322 women (mean age: 50.5, *SD* = 18.3), and *n* = 4 participants identifying as diverse. Detailed sample characteristics are provided in **Table 1**. **Figure 1** presents a flowchart outlining the sampling procedure and reasons for nonparticipation. For cross-cohort analyses, a comparison sample was drawn from a representative German survey collected in November/December 2009 (*N* = 2,520), identical to the normative sample reported by Franke et al. (34). The present primary dataset has previously been used for the psychometric evaluation of the PHQ-9 (30) and the GAD-7 (32).

**Table 1:** Demographic characteristics of the study sample.

Characteristic	Men (*N* = 1193)	Women (*N* = 1322)	Diverse (*N* = 4)	Total (*N* = 2519)
Age (years)				
Mean (SD)	50.1 (17.7)	50.5 (18.3)	44.8 (26.5)	50.3 (18.1)
Median [min, max]	52.0 [16.0, 96.0]	51.0 [16.0, 96.0]	41.5 [21.0, 75.0]	51.0 [16.0, 96.0]
Age categories				
16-24	102 (8.5%)	125 (9.5%)	2 (50.0%)	229 (9.1%)
25-34	190 (15.9%)	174 (13.2%)	0 (0%)	364 (14.5%)
35-44	174 (14.6%)	225 (17.0%)	0 (0%)	399 (15.8%)
45-54	195 (16.3%)	216 (16.3%)	0 (0%)	411 (16.3%)
55-64	244 (20.5%)	243 (18.4%)	1 (25.0%)	488 (19.4%)
65-74	190 (15.9%)	200 (15.1%)	0 (0%)	390 (15.5%)
75+	98 (8.2%)	139 (10.5%)	1 (25.0%)	238 (9.4%)
Migration background				
no	1151 (96.5%)	1271 (96.1%)	3 (75.0%)	2425 (96.3%)
yes	42 (3.5%)	48 (3.6%)	1 (25.0%)	91 (3.6%)
Missing	0 (0%)	3 (0.2%)	0 (0%)	3 (0.1%)
Marital status				
married/living together	547 (45.9%)	527 (39.9%)	2 (50.0%)	1076 (42.7%)
married/separated	40 (3.4%)	25 (1.9%)	0 (0%)	65 (2.6%)
single	398 (33.4%)	357 (27.0%)	2 (50.0%)	757 (30.1%)
divorced	143 (12.0%)	227 (17.2%)	0 (0%)	370 (14.7%)
widowed	62 (5.2%)	181 (13.7%)	0 (0%)	243 (9.6%)
Missing	3 (0.3%)	5 (0.4%)	0 (0%)	8 (0.3%)
Living with partner				
Yes	729 (61.1%)	737 (55.7%)	2 (50.0%)	1468 (58.3%)
No	444 (37.2%)	565 (42.7%)	2 (50.0%)	1011 (40.1%)
Missing	20 (1.7%)	20 (1.5%)	0 (0%)	40 (1.6%)

### 2.3 Instruments

#### 2.3.1 Brief symptom inventory-18

The BSI-18 [ (10), German version (9):] is a brief self-report questionnaire assessing psychological distress during the past seven days. The BSI-18 comprises 18 items covering three symptom dimensions: somatization (SOM; 6 items, e.g. faintness or dizziness, pains in heart or chest), depression (DEP; 6 items, e.g. feeling blue, feelings of worthlessness), and anxiety (ANX; 6 items, e.g. nervousness or shakiness, spells of terror or panic). Items are rated on a five-point Likert scale ranging from 0 (not at all) to 4 (extremely). Each subscale yields a sum score ranging from 0 to 24, and the Global Severity Index (GSI) is computed as the sum of all 18 items, yielding a total score between 0 and 72, with higher scores indicating greater psychological distress.

#### 2.3.2 Patient health questionnaire-9

The PHQ-9 [ (36), German version (37):] is a nine-item self-report measure of depressive symptom severity over the past two weeks. Items are rated on a four-point scale from 0 (not at all) to 3 (nearly every day), yielding a total score ranging from 0 to 27. A cut-off of ≥ 10 is commonly used to indicate at least moderate depressive symptoms. Internal consistency in the present study was omega = 0.929, 95% CI [0.920, 0.938], α = 0.901, 95% CI [0.889, 0.910] [see Kliem et al. (30) for details].

#### 2.3.3 Generalized anxiety disorder-7

The GAD-7 [ (38), German version (39):] is a seven-item self-report measure of anxiety symptom severity over the past two weeks. Items are rated on a four-point scale from 0 (not at all) to 3 (nearly every day), yielding a total score ranging from 0 to 21. A cut-off of ≥ 10 is commonly used to indicate at least moderate anxiety. Internal consistency in the present study was omega = 0.917, 95% CI [0.908, 0.926], α = 0.902, 95% CI [0.892, 0.912] [see Kliem et al. (32) for details].

#### 2.3.4 Chalder fatigue questionnaire-11

The CFQ-11 [ (40), German version (41):] is an 11-item self-report measure of fatigue severity over the past month. Items are rated on a four-point scale from 0 (less than usual) to 3 (much more than usual), yielding a total score ranging from 0 to 33. The CFQ-11 comprises two subscales: physical fatigue (items 1-7; e.g., *do you have problems with tiredness*)? and mental fatigue (items 8-11; e.g., *do you have difficulty concentrating*)?. Internal consistency in the present study was omega = 0.978, 95% CI [0.969, 0.990], α = 0.939, 95% CI [0.934, 0.944]. The CFQ-11 served as the criterion measure for the somatization dimension because it was the only dedicated measure of somatic symptom burden included in the survey. A broad somatic symptom measure such as the PHQ-15 was not administered, as the multi-thematic survey combined instruments contributed by several research groups. This availability-driven choice should be considered when interpreting the convergent validity findings (see Limitations).

### 2.4 Statistical analysis

All analyses were conducted in R (42).

#### 2.4.1 Missing data

Missing values were imputed using predictive mean matching within the mice framework (43) with a single imputation (m = 1, maxit = 50, seed = 777). A single completed dataset was used because item-level missingness was minimal, because the smoothed percentile norms require a single completed dataset, and to retain consistency with the published companion analyses of the PHQ-9 (30) and GAD-7 (32) based on the same sample. The imputation model included all BSI-18 items, PHQ-9 items, GAD-7 items, and sociodemographic predictors (age, gender, education, income, household size).

#### 2.4.2 Item analysis and internal consistency

Item characteristics were examined using descriptive statistics, item difficulty indices (percentage of maximum score), corrected item-total correlations, and Cronbach’s alpha if-item-deleted. Inter-item correlations were computed for the full 18-item set. To account for potential issues arising from unmet assumptions in the calculation of coefficient alpha (44), internal consistency was assessed using both McDonald’s omega, computed from standardized CFA factor loadings via the semTools package (45), and Cronbach’s alpha. 95% confidence intervals for both coefficients were obtained via percentile bootstrap with *B* = 1,000 resamples. Reliability estimates were computed separately for the total sample, men, and women. Item characteristics were obtained using the R package psych (46).

#### 2.4.3 Confirmatory factor analysis

Factorial validity was examined using confirmatory factor analysis (CFA) with the lavaan package (47). Given the ordered categorical nature of the items and pronounced floor effects, WLSMV estimation was used (48). Three models were compared: (i) a unidimensional model (M1) in which all 18 items loaded on a single general distress factor, (ii) a correlated three-factor model (M2) corresponding to the SOM, DEP, and ANX subscales, and (iii) a higher-order model (M3) with a second-order general distress factor. In M3, the second-order loadings were constrained to equality to prevent boundary estimates resulting from the high first-order factor correlations. Model fit was evaluated using the following criteria: CFI and TLI ≥ 0.95 indicate good fit, RMSEA ≤ 0.06 indicates close fit, and SRMR ≤ 0.08 indicates adequate fit (49). Because WLSMV estimation applies a mean and variance correction to the chi-square statistic, all fit indices reported are the scaled variants (i.e., robust CFI, robust TLI, robust RMSEA with 90% CI). SRMR does not have a scaled variant under WLSMV and is reported as the standard estimate. Model comparison was performed using the scaled chi-square difference test.

#### 2.4.4 Gender differences

Gender differences were evaluated using permutation tests (10,000 resamples). Effect sizes were quantified as Cohen’s *d* for sum scores and Cliff’s delta for individual items, each with 95% confidence intervals (50).

#### 2.4.5 Construct validity

Construct validity was evaluated through latent correlations between the BSI-18 factors and external criterion measures using the WLSMV estimator. For each criterion measure, a separate structural equation model was estimated in which the BSI-18 was specified as the correlated three-factor model (M2) and the criterion as a single latent factor. Correlations involving the GSI were estimated analogously, with the BSI-18 specified as the unidimensional model (M1). The following convergent validity hypotheses were formulated: (a) DEP should correlate most highly with the PHQ-9, (b) ANX with the GAD-7, and (c) SOM with the CFQ-11. Evidence for discriminant validity was expected from lower off-diagonal correlations. 95% confidence intervals were obtained via percentile bootstrap (*B* = 1,000).

#### 2.4.6 Measurement invariance

Measurement invariance (MI) was tested using multi-group CFA (MGCFA) following the procedure suggested by Wu and Estabrook (51) for ordered categorical data. The correlated three-factor model (M2) was used with theta parameterization. Five nested models were tested: (i) configural invariance (unconstrained except for identification constraints), (ii) threshold invariance (equal thresholds across groups), (iii) weak invariance (equal factor loadings), (iv) strong invariance (equal intercepts), and (v) full invariance (equal residual variances). The parameter constraints for each model are visualized in **Supplementary Figure 1** and described in detail in **Supplementary Table 1**. Chen’s (52) cut-off criteria were used, with a change of < -0.010 in CFI and a change of ≥ 0.015 in RMSEA indicating non-invariance. MI was tested across four grouping variables: gender (men vs. women), age (below vs. above median), and clinical screening status defined by PHQ-9 ≥ 10 (36) as well as GAD-7 ≥ 10 (38). Age was dichotomized at the sample median because stable threshold estimation under WLSMV requires large groups with all response categories occupied. Given the pronounced floor effects of the items, finer age strata would have produced empty response categories in low-endorsement items (cf. footnote 1). This corresponds to the procedure used in the PHQ-9 (30) and GAD-7 (32) invariance analyses based on the same sample. Sparse response categories were collapsed prior to analysis (categories 3 and 4 were merged) to ensure model identification across all subgroups. The *n* = 4 participants identifying as diverse were excluded from gender-specific comparisons.

#### 2.4.7 Population norms

Smoothed cumulative percentiles were estimated using shape-constrained additive models (SCAM), ensuring monotonically non-decreasing percentile functions. 95% confidence intervals were obtained via percentile bootstrap (*B* = 1,000). Norms are presented for the total sample and stratified by gender and age group (16-24, 25-34, 35-44, 45-54, 55-64, 65-74, 75+). All norms are based on unweighted data.

#### 2.4.8 Cross-cohort comparison

Item-level and total score comparisons were conducted between the present sample and the 2009 normative sample [ (34); *N* = 2,520, age-filtered to ≥ 16: *N* = 2,473). Effect sizes were quantified as Cohen’s *d* for sum scores and Cliff’s delta for items. Permutation tests (10,000 resamples) were used for significance testing.

## 3 Results

### 3.1 Item characteristics and reliability

The proportion of missing data was low (0.4% to 0.6% per item). After imputation, descriptive statistics for all 18 items are presented in **Table 2**. Item means ranged from 0.06 (item 17, thoughts of ending your life) to 0.46 (item 14, feeling hopeless about future), reflecting pronounced floor effects. Item difficulty indices (Pi) ranged from 1.53% (item 17) to 11.44% (item 14). Endorsement rates for the lowest response category (0 = not at all) ranged from 85% to 96%, indicating that the majority of the general population does not endorse most symptoms. Skewness values exceeded 2 for all items and kurtosis exceeded 7 for the majority, confirming substantial departures from normality and justifying the use of the WLSMV estimator. Mean subscale sum scores were *M* = 1.21 (*SD* = 2.53) for SOM, *M* = 1.89 (*SD* = 3.38) for DEP, *M* = 1.25 (*SD* = 2.54) for ANX, and *M* = 4.35 (*SD* = 7.62) for the GSI.

**Table 2:** Item characteristics of the BSI-18 (N = 2,519).

Nr	Sub	Item	*M*	*SD*	*Skew*	*Kurt*	*Pi*	*r_it_*	*α^-1^*	*λ*
1	SOM	Faintness/dizziness	0.15	0.48	3.78	16.66	3.86	0.61	0.93	0.80
4	SOM	Pains in heart/chest	0.16	0.49	3.55	14.34	4.10	0.62	0.93	0.81
7	SOM	Nausea/upset stomach	0.23	0.59	3.10	11.25	5.83	0.52	0.93	0.68
10	SOM	Trouble getting breath	0.17	0.55	3.85	16.08	4.21	0.60	0.93	0.81
13	SOM	Numbness/tingling	0.20	0.58	3.62	14.72	4.99	0.59	0.93	0.75
16	SOM	Feeling weak	0.29	0.69	2.81	8.21	7.16	0.69	0.93	0.83
		**SOM total**	1.21	2.53	3.20	12.79				
2	DEP	No interest in things	0.38	0.73	2.28	5.53	9.48	0.72	0.93	0.83
5	DEP	Feeling lonely	0.45	0.87	2.14	4.22	11.33	0.66	0.93	0.79
8	DEP	Feeling blue	0.29	0.69	2.82	8.41	7.27	0.74	0.93	0.87
11	DEP	Feelings of worthlessness	0.25	0.68	3.24	10.97	6.16	0.73	0.93	0.88
14	DEP	Feeling hopeless	0.46	0.88	2.15	4.28	11.44	0.69	0.93	0.82
17	DEP	Thoughts of ending life	0.06	0.34	6.87	54.58	1.53	0.53	0.93	0.82
		**DEP total**	1.89	3.38	2.65	8.04				
3	ANX	Nervousness/shakiness	0.25	0.60	2.93	9.75	6.20	0.73	0.93	0.86
6	ANX	Feeling tense/keyed up	0.41	0.73	1.97	3.93	10.25	0.61	0.93	0.74
9	ANX	Suddenly scared	0.11	0.43	4.60	24.61	2.85	0.65	0.93	0.85
12	ANX	Spells of terror/panic	0.10	0.41	4.96	28.70	2.55	0.68	0.93	0.90
15	ANX	Feeling restless	0.22	0.62	3.46	13.31	5.51	0.65	0.93	0.80
18	ANX	Feeling fearful	0.16	0.49	3.70	15.30	3.94	0.68	0.93	0.86
		**ANX total**	1.25	2.54	3.22	12.97				
		**GSI total**	4.35	7.62	3.00	11.53				

Items rated 0–4. SOM, somatization; DEP, depression; ANX, anxiety; GSI, Global Severity Index. Pi = difficulty index. rit = corrected item-total correlation (GSI). λ = factor loading (M1, WLSMV). Bold values indicate the subscale (SOM, DEP, ANX) and GSI total scores.

Corrected item-total correlations ranged from 0.52 (item 7, nausea/upset stomach) to 0.74 (item 8, feeling blue), indicating adequate to good item discrimination. Item 17 had the lowest mean (*M* = 0.06, *SD* = 0.34) and highest kurtosis (54.58), reflecting the very low endorsement rate of suicidal ideation in the general population (Pi = 1.53%). Despite these extreme distributional properties, item 17 showed adequate item-total correlation (*r*_it_ = 0.53) and a high factor loading (λ = 0.82). Standardized factor loadings from the unidimensional CFA (M1, see 3.2) ranged from 0.68 (item 7: nausea/upset stomach) to 0.90 (item 12: spells of terror/panic).

Internal consistency coefficients are presented in **Supplementary Table 2**. McDonald’s omega for the GSI was 0.966, 95% CI [0.961, 0.974]; Cronbach’s alpha was 0.932, 95% CI [0.923, 0.941]. For the subscales, omega ranged from 0.855 (SOM) to 0.893 (DEP). Reliability was comparable across gender subgroups (**Supplementary Table 2**). Alpha if-item-deleted remained at 0.93 for all items, indicating that no single item substantially reduced the internal consistency of the GSI.

### 3.2 Confirmatory factor analysis

**Supplementary Table 3** presents fit indices for all models. The unidimensional model (M1) showed acceptable fit: χ^2^(135) = 1525, CFI = 0.961, TLI = 0.955, RMSEA = 0.064 (90% CI [0.061, 0.067]), SRMR = 0.054. The correlated three-factor model (M2) showed good fit: χ^2^(132) = 644, CFI = 0.986, TLI = 0.983, RMSEA = 0.039 (90% CI [0.036, 0.042]), SRMR = 0.034. The difference between M1 and M2 was statistically significant (scaled chi-square difference test, *p* < 0.001), confirming that the three-factor structure provides a substantially better representation of the data. The higher-order model (M3) also showed good fit: χ^2^(134) = 732, CFI = 0.983, TLI = 0.981, RMSEA = 0.042 (90% CI [0.039, 0.045]), SRMR = 0.039. The practical difference between M2 and M3 was negligible (Δ-CFI = 0.003). All fit indices for both M2 and M3 were within or exceeded the recommended cut-off criteria. In M2, all standardized first-order factor loadings exceeded the minimum threshold of 0.40, ranging from 0.73 (item 7: nausea/upset stomach, on SOM) to 0.92 (item 12: spells of terror/panic, on ANX; **Supplementary Table 4**). Loadings were highest for the ANX factor (0.76 to 0.92), followed by DEP (0.82 to 0.91) and SOM (0.73 to 0.88). Latent factor correlations in M2 were high: *r* = 0.824 (SOM-DEP), *r* = 0.895 (SOM-ANX), *r* = 0.913 (DEP-ANX). Second-order loadings in M3 indicated that all three factors were well represented by the general distress factor: SOM = 0.920, DEP = 0.934, ANX = 0.961. These results contrast markedly with Franke et al. (34), who obtained CFI = 0.55 using the ADF estimator. The present findings confirm that the poor fit was attributable to the choice of estimator (48), consistent with Petrowski et al. (53). Based on these results, the correlated three-factor model (M2) was retained as the final model: measurement invariance testing and the latent validity correlations of the three subscales were based on the M2 specification, whereas the latent correlations involving the GSI were estimated from the unidimensional specification (M1; see Section 2.4.5).

### 3.3 Gender differences

Women scored significantly higher than men on the GSI [*M* = 4.83, *SD* = 7.78 vs. *M* = 3.78, *SD* = 7.31; *d* = -0.14, 95% CI (-0.22, -0.06)] and on all three subscales: SOM [*M* = 1.33 vs. 1.06; *d* = -0.11, 95% CI (-0.18, -0.03), *p* = 0.008], DEP [*M* = 2.09 vs. 1.65; *d* = -0.13, 95% CI (-0.21, -0.05), *p* = 0.001], and ANX [*M* = 1.41 vs. 1.07; *d* = -0.14, 95% CI (-0.21, -0.06), *p* = 0.001]. All effects were small according to conventional criteria (50).

At the item level, 12 of 18 items showed statistically significant gender differences (permutation test, *B* = 10,000). The largest effects were observed for item 5 (feeling lonely; δ = -0.10, 95% CI (-0.13, -0.06)], item 3 (nervousness/shakiness; δ = -0.07), and item 6 (feeling tense/keyed up; δ = -0.06). Six items did not differ significantly between genders: item 4 (pains in heart/chest; *p* = 0.933), item 10 (trouble getting breath; *p* = 0.213), item 11 (feelings of worthlessness; *p* = 0.247), item 13 (numbness/tingling; *p* = 0.111), item 15 (feeling restless; *p* = 0.801), and item 16 (feeling weak; *p* = 0.059). Item 17 (thoughts of ending your life) showed virtually identical means (*M* = 0.06 for all genders; *p* = 0.907). Complete results are presented in **Supplementary Table 5**.

### 3.4 Construct validity

Latent correlations are presented in **Supplementary Table 6**. The convergent validity hypotheses were confirmed for depression and anxiety: DEP correlated most highly with the PHQ-9 [r = 0.912, 95% CI (0.893, 0.932)] and ANX with the GAD-7 [r = 0.889, 95% CI (0.867, 0.911)]. In both cases, the convergent correlation exceeded all off-diagonal correlations of the respective row and column. For somatization, the hypothesis was only partially supported: SOM correlated substantially with the CFQ-11 [r = 0.580, 95% CI (0.533, 0.630)], but more strongly with the PHQ-9 [r = 0.799, 95% CI (0.764, 0.835)], and the CFQ-11 correlated as strongly with DEP (r = 0.592) as with SOM. The GSI correlated highly with all criterion measures, with the highest correlation observed for the PHQ-9 (r = 0.899, 95% CI [0.879, 0.919]), followed by the GAD-7 [r = 0.873, 95% CI (0.853, 0.893)] and the CFQ-11 [r = 0.589, 95% CI (0.546, 0.635)]. Manifest correlations of the three convergent pairs showed the same ordering at a uniformly lower magnitude (r = 0.458 to 0.769). The attenuation relative to the latent correlations reflects the disattenuation for measurement error inherent in the WLSMV structural models.

### 3.5 Measurement invariance

Measurement invariance results are presented in **Supplementary Table 7**. Across gender, the configural model showed good fit [χ^2^(264) = 693.0, CFI = 0.988, RMSEA = 0.036, 90% CI (0.033, 0.039)]. Each successive constraint, threshold (Δ-CFI = 0.000), weak (Δ-CFI = 0.001), strong (Δ-CFI = 0.001), and full invariance (Δ-CFI = 0.001), resulted in negligible changes. Notably, fit indices improved rather than deteriorated across levels (RMSEA decreased from 0.036 to 0.027), indicating that the more constrained models provided a more parsimonious and equally adequate representation of the data.

Across age (median split at 53 years), full invariance was likewise supported. The configural model fit well (χ^2^(264) = 724.6, CFI = 0.987, RMSEA = 0.037), and successive restrictions produced Δ-CFI values between 0.000 and 0.001 (Δ-RMSEA from -0.001 to -0.002).

Measurement invariance was further examined across clinical screening status using established cut-off scores. For depression caseness (PHQ-9 ≥ 10), the configural model showed good fit (χ^2^(264) = 692.2, CFI = 0.976, RMSEA = 0.036), and full invariance was supported (Δ-CFI from -0.001 to 0.002; Δ-RMSEA from -0.001 to -0.002). For anxiety caseness (GAD-7 ≥ 10), an analogous pattern emerged (configural: χ^2^(264) = 664.9, CFI = 0.982, RMSEA = 0.035; full: Δ-CFI from -0.001 to 0.002). These results indicate that the BSI-18 measures the same constructs with the same metric properties in individuals with and without clinically relevant symptom levels.^1^.

### 3.6 Population norms

Smoothed cumulative percentiles for the GSI, SOM, DEP, and ANX are presented in **Tables 3**–**6** for the total sample and by gender, and in **Supplementary Tables 8–15** stratified by gender and age group. For the total sample, 42.3% [95% CI (40.3, 44.2)] obtained a GSI of 0. This proportion was higher in men (46.5%) than in women (38.6%) and decreased with age, with the lowest floor proportion in the 75+ group (men: 24.3%, women: 18.1%). A GSI score of 10 corresponded to the 87.2nd percentile [95% CI (85.9, 88.3)] in the total sample, the 89.8th percentile in men, and the 84.9th percentile in women. Floor effects were more pronounced for the subscales than for the GSI. For SOM, 65.3% of the total sample scored 0 (men: 68.5%, women: 62.4%), and a score of 4 corresponded to the 91.2nd percentile. For ANX, 61.3% scored 0 (men: 65.0%, women: 58.1%), with a score of 4 corresponding to the 91.6th percentile. For DEP, 54.4% scored 0 (men: 58.2%, women: 51.1%), and a score of 4 corresponded to the 85.8th percentile. Gender-specific norms indicated that men scored lower across all scales: for the GSI, a score of 4 corresponded to the 76.1st percentile in men but only the 69.6th percentile in women. Age-specific norms for seven age groups are provided in **Supplementary Tables 8–15** for men and women separately.

**Table 3:** Smoothed cumulative percentiles for the GSI (0–72), total sample and by gender.

Score	Total [95% CI]	Men [95% CI]	Women [95% CI]
0	42.3 [40.3, 44.2]	46.5 [43.4, 49.5]	38.6 [36.0, 41.3]
1	52.2 [50.5, 53.8]	56.2 [53.7, 58.8]	48.8 [46.3, 51.2]
2	60.5 [58.9, 62.0]	64.2 [62.0, 66.7]	57.2 [54.8, 59.5]
3	67.2 [65.7, 68.7]	70.8 [68.5, 73.2]	64.1 [61.8, 66.4]
4	72.6 [71.2, 74.2]	76.1 [73.8, 78.4]	69.6 [67.4, 72.0]
5	76.9 [75.4, 78.5]	80.2 [78.0, 82.4]	74.0 [71.8, 76.2]
6	80.2 [78.8, 81.7]	83.4 [81.3, 85.4]	77.5 [75.2, 79.6]
7	82.7 [81.4, 84.1]	85.7 [83.8, 87.7]	80.1 [77.9, 82.2]
8	84.6 [83.3, 85.9]	87.5 [85.7, 89.3]	82.1 [80.0, 84.1]
9	86.0 [84.7, 87.2]	88.8 [87.1, 90.4]	83.6 [81.7, 85.6]
10	87.2 [85.9, 88.3]	89.8 [88.2, 91.4]	84.9 [83.1, 86.7]
11	88.2 [87.0, 89.3]	90.6 [89.1, 92.2]	86.1 [84.3, 87.8]
12	89.2 [88.0, 90.2]	91.5 [90.0, 93.0]	87.2 [85.5, 88.9]
13	90.1 [89.0, 91.1]	92.2 [90.8, 93.6]	88.2 [86.6, 89.9]
14	90.9 [89.9, 92.0]	92.9 [91.5, 94.3]	89.2 [87.6, 90.8]
15	91.8 [90.8, 92.7]	93.5 [92.2, 94.8]	90.2 [88.7, 91.7]
16	92.5 [91.6, 93.4]	94.1 [92.9, 95.3]	91.1 [89.7, 92.5]
17	93.2 [92.3, 94.1]	94.6 [93.4, 95.7]	92.0 [90.6, 93.3]
18	93.8 [93.0, 94.7]	95.1 [94.0, 96.1]	92.8 [91.5, 94.0]
19	94.4 [93.6, 95.3]	95.5 [94.4, 96.5]	93.5 [92.2, 94.7]
20	95.0 [94.2, 95.8]	95.9 [94.8, 96.9]	94.2 [92.9, 95.4]
21	95.5 [94.7, 96.3]	96.3 [95.2, 97.3]	94.8 [93.6, 95.9]
22	95.9 [95.1, 96.7]	96.6 [95.6, 97.6]	95.3 [94.2, 96.4]
23	96.3 [95.6, 97.0]	96.9 [95.9, 97.8]	95.8 [94.7, 96.8]
24	96.6 [96.0, 97.3]	97.2 [96.2, 98.1]	96.2 [95.2, 97.2]
25	97.0 [96.3, 97.6]	97.4 [96.5, 98.3]	96.6 [95.6, 97.5]
26	97.2 [96.6, 97.8]	97.6 [96.8, 98.4]	96.9 [96.0, 97.8]
27	97.5 [96.9, 98.0]	97.8 [97.0, 98.6]	97.2 [96.3, 98.0]
28	97.7 [97.1, 98.2]	98.0 [97.2, 98.7]	97.5 [96.6, 98.2]
29	97.9 [97.4, 98.4]	98.2 [97.4, 98.8]	97.7 [96.9, 98.4]
30	98.1 [97.6, 98.6]	98.3 [97.6, 98.9]	98.0 [97.2, 98.6]
31	98.3 [97.8, 98.7]	98.5 [97.7, 99.1]	98.2 [97.4, 98.8]
32	98.4 [97.9, 98.9]	98.6 [97.9, 99.2]	98.4 [97.7, 98.9]
33	98.6 [98.1, 99.0]	98.7 [98.0, 99.2]	98.5 [97.9, 99.1]
34	98.7 [98.3, 99.1]	98.8 [98.2, 99.3]	98.7 [98.1, 99.2]
35	98.8 [98.4, 99.2]	98.9 [98.3, 99.4]	98.9 [98.3, 99.3]
36	99.0 [98.6, 99.3]	99.0 [98.4, 99.5]	99.0 [98.5, 99.4]
37	99.1 [98.7, 99.4]	99.1 [98.6, 99.5]	99.2 [98.7, 99.5]
38	99.2 [98.9, 99.5]	99.2 [98.7, 99.6]	99.3 [98.9, 99.6]
39	99.3 [99.0, 99.5]	99.2 [98.8, 99.6]	99.4 [99.0, 99.7]
40	99.4 [99.1, 99.6]	99.3 [98.9, 99.7]	99.5 [99.1, 99.7]
41	99.4 [99.2, 99.7]	99.3 [98.9, 99.7]	99.6 [99.3, 99.8]
42	99.5 [99.2, 99.7]	99.4 [99.0, 99.8]	99.6 [99.4, 99.9]
43	99.6 [99.3, 99.8]	99.4 [99.1, 99.8]	99.7 [99.4, 99.9]
44	99.6 [99.4, 99.8]	99.5 [99.1, 99.8]	99.8 [99.5, 99.9]
45	99.7 [99.4, 99.9]	99.5 [99.2, 99.9]	99.8 [99.5, >99.9]
46	99.7 [99.5, 99.9]	99.6 [99.3, 99.9]	99.8 [99.6, >99.9]
47	99.7 [99.5, 99.9]	99.6 [99.3, 99.9]	99.9 [99.6, >99.9]
48	99.8 [99.6, 99.9]	99.6 [99.4, 99.9]	99.9 [99.7, >99.9]
49	99.8 [99.6, 99.9]	99.7 [99.4, 99.9]	99.9 [99.7, >99.9]
50	99.8 [99.6, >99.9]	99.7 [99.4, 99.9]	99.9 [99.7, >99.9]
51	99.8 [99.7, >99.9]	99.7 [99.5, 99.9]	99.9 [99.7, >99.9]
52	99.8 [99.7, >99.9]	99.7 [99.5, >99.9]	99.9 [99.8, >99.9]
53	99.9 [99.7, >99.9]	99.8 [99.5, >99.9]	99.9 [99.8, >99.9]
54	99.9 [99.7, >99.9]	99.8 [99.6, >99.9]	99.9 [99.8, >99.9]
55	99.9 [99.8, >99.9]	99.8 [99.6, >99.9]	>99.9 [99.8, >99.9]
56	99.9 [99.8, >99.9]	99.8 [99.6, >99.9]	>99.9 [99.9, >99.9]
57	99.9 [99.8, >99.9]	99.8 [99.6, >99.9]	>99.9 [99.9, >99.9]
58	99.9 [99.8, >99.9]	99.9 [99.7, >99.9]	>99.9 [99.9, >99.9]
59	99.9 [99.8, >99.9]	99.9 [99.7, >99.9]	>99.9 [99.9, >99.9]
60	99.9 [99.9, >99.9]	99.9 [99.7, >99.9]	>99.9 [99.9, >99.9]
61	99.9 [99.9, >99.9]	99.9 [99.7, >99.9]	>99.9 [>99.9, >99.9]
62	>99.9 [99.9, >99.9]	99.9 [99.7, >99.9]	>99.9 [>99.9, >99.9]
63	>99.9 [99.9, >99.9]	99.9 [99.8, >99.9]	>99.9 [>99.9, >99.9]
64	>99.9 [99.9, >99.9]	99.9 [99.8, >99.9]	>99.9 [>99.9, >99.9]
65	>99.9 [99.9, >99.9]	>99.9 [99.8, >99.9]	>99.9 [>99.9, >99.9]
66	>99.9 [99.9, >99.9]	>99.9 [99.8, >99.9]	>99.9 [>99.9, >99.9]
67	>99.9 [99.9, >99.9]	>99.9 [99.8, >99.9]	>99.9 [>99.9, >99.9]
68	>99.9 [99.9, >99.9]	>99.9 [99.8, >99.9]	>99.9 [>99.9, >99.9]
69	>99.9 [99.9, >99.9]	>99.9 [99.8, >99.9]	>99.9 [>99.9, >99.9]
70	>99.9 [99.9, >99.9]	>99.9 [99.8, >99.9]	>99.9 [>99.9, >99.9]
71	>99.9 [99.9, >99.9]	>99.9 [99.8, >99.9]	>99.9 [>99.9, >99.9]
72	>99.9 [>99.9, >99.9]	>99.9 [99.8, >99.9]	>99.9 [>99.9, >99.9]

Percentile bootstrap CIs (B = 1,000).

**Table 4:** Smoothed cumulative percentiles for SOM (0–24), total sample and by gender.

Score	Total [95% CI]	Men [95% CI]	Women [95% CI]
0	65.3 [63.4, 67.0]	68.5 [66.0, 71.2]	62.4 [60.0, 65.0]
1	77.0 [75.5, 78.6]	79.8 [77.8, 81.8]	74.5 [72.4, 76.9]
2	84.2 [82.8, 85.5]	86.5 [84.7, 88.4]	82.2 [80.2, 84.2]
3	88.4 [87.3, 89.6]	90.3 [88.7, 91.8]	86.8 [85.1, 88.5]
4	91.2 [90.2, 92.3]	92.7 [91.3, 94.1]	89.9 [88.2, 91.5]
5	93.3 [92.4, 94.3]	94.5 [93.4, 95.8]	92.3 [90.9, 93.7]
6	94.9 [94.1, 95.7]	95.9 [94.8, 96.9]	94.1 [92.9, 95.3]
7	96.1 [95.4, 96.8]	96.9 [95.9, 97.8]	95.5 [94.5, 96.6]
8	97.1 [96.4, 97.7]	97.6 [96.7, 98.4]	96.7 [95.8, 97.6]
9	97.8 [97.3, 98.3]	98.1 [97.4, 98.8]	97.6 [96.9, 98.3]
10	98.4 [97.9, 98.8]	98.5 [97.7, 99.0]	98.4 [97.7, 98.9]
11	98.8 [98.4, 99.2]	98.8 [98.1, 99.3]	98.9 [98.3, 99.4]
12	99.1 [98.7, 99.4]	99.0 [98.4, 99.5]	99.2 [98.8, 99.6]
13	99.3 [99.0, 99.6]	99.2 [98.7, 99.6]	99.5 [99.0, 99.8]
14	99.5 [99.2, 99.7]	99.4 [98.9, 99.7]	99.6 [99.3, 99.9]
15	99.6 [99.4, 99.8]	99.5 [99.1, 99.8]	99.7 [99.5, >99.9]
16	99.7 [99.6, 99.9]	99.6 [99.3, 99.9]	99.8 [99.7, >99.9]
17	99.8 [99.7, 99.9]	99.7 [99.4, 99.9]	99.9 [99.8, >99.9]
18	99.9 [99.8, >99.9]	99.8 [99.5, >99.9]	99.9 [99.9, >99.9]
19	99.9 [99.8, >99.9]	99.8 [99.6, >99.9]	>99.9 [99.9, >99.9]
20	99.9 [99.9, >99.9]	99.9 [99.7, >99.9]	>99.9 [>99.9, >99.9]
21	>99.9 [99.9, >99.9]	99.9 [99.8, >99.9]	>99.9 [>99.9, >99.9]
22	>99.9 [99.9, >99.9]	>99.9 [99.8, >99.9]	>99.9 [>99.9, >99.9]
23	>99.9 [>99.9, >99.9]	>99.9 [99.9, >99.9]	>99.9 [>99.9, >99.9]
24	>99.9 [>99.9, >99.9]	>99.9 [99.9, >99.9]	>99.9 [>99.9, >99.9]

Percentile bootstrap CIs (B = 1,000).

**Table 5:** Smoothed cumulative percentiles for DEP (0–24), total sample and by gender.

Score	Total [95% CI]	Men [95% CI]	Women [95% CI]
0	54.4 [52.4, 56.5]	58.2 [55.7, 61.1]	51.1 [48.1, 54.0]
1	67.8 [66.1, 69.5]	71.5 [69.2, 74.0]	64.5 [62.0, 67.0]
2	76.4 [74.8, 78.1]	79.8 [77.7, 82.1]	73.4 [71.1, 75.6]
3	82.0 [80.5, 83.3]	84.9 [83.1, 86.9]	79.3 [77.2, 81.3]
4	85.8 [84.5, 87.0]	88.2 [86.5, 90.0]	83.6 [81.6, 85.5]
5	88.7 [87.5, 89.8]	90.6 [89.0, 92.2]	87.0 [85.1, 88.7]
6	90.9 [89.8, 92.0]	92.3 [90.9, 93.8]	89.7 [88.0, 91.2]
7	92.6 [91.6, 93.6]	93.7 [92.3, 95.0]	91.8 [90.2, 93.2]
8	94.0 [93.1, 94.9]	94.9 [93.6, 96.0]	93.4 [91.9, 94.6]
9	95.2 [94.4, 96.0]	95.9 [94.8, 96.9]	94.6 [93.3, 95.7]
10	96.1 [95.4, 96.8]	96.7 [95.8, 97.6]	95.6 [94.5, 96.6]
11	96.9 [96.3, 97.5]	97.4 [96.5, 98.2]	96.5 [95.5, 97.4]
12	97.6 [97.0, 98.1]	97.9 [97.1, 98.6]	97.3 [96.4, 98.0]
13	98.1 [97.6, 98.5]	98.3 [97.6, 98.9]	97.9 [97.1, 98.6]
14	98.5 [98.1, 98.9]	98.6 [98.0, 99.2]	98.5 [97.8, 99.1]
15	98.9 [98.5, 99.2]	98.9 [98.3, 99.4]	98.9 [98.3, 99.4]
16	99.1 [98.8, 99.4]	99.1 [98.6, 99.5]	99.2 [98.8, 99.6]
17	99.3 [99.1, 99.6]	99.3 [98.9, 99.7]	99.4 [99.1, 99.8]
18	99.5 [99.3, 99.7]	99.4 [99.1, 99.8]	99.6 [99.4, 99.9]
19	99.7 [99.5, 99.8]	99.6 [99.3, 99.8]	99.7 [99.5, >99.9]
20	99.8 [99.6, 99.9]	99.7 [99.4, 99.9]	99.8 [99.7, >99.9]
21	99.9 [99.7, >99.9]	99.8 [99.6, >99.9]	99.9 [99.8, >99.9]
22	99.9 [99.8, >99.9]	99.9 [99.7, >99.9]	>99.9 [99.9, >99.9]
23	>99.9 [99.9, >99.9]	>99.9 [99.9, >99.9]	>99.9 [99.9, >99.9]
24	>99.9 [>99.9, >99.9]	>99.9 [>99.9, >99.9]	>99.9 [>99.9, >99.9]

Percentile bootstrap CIs (B = 1,000).

**Table 6:** Smoothed cumulative percentiles for ANX (0–24), total sample and by gender.

Score	Total [95% CI]	Men [95% CI]	Women [95% CI]
0	61.3 [59.4, 63.1]	65.0 [62.1, 67.5]	58.1 [55.4, 60.8]
1	76.2 [74.7, 77.7]	79.2 [77.0, 81.1]	73.6 [71.2, 75.6]
2	84.7 [83.3, 86.0]	87.4 [85.5, 89.1]	82.4 [80.2, 84.2]
3	89.1 [87.9, 90.2]	91.5 [90.0, 92.8]	86.9 [84.9, 88.5]
4	91.6 [90.6, 92.6]	93.7 [92.3, 94.9]	89.7 [88.1, 91.2]
5	93.3 [92.4, 94.2]	95.0 [93.7, 96.1]	91.8 [90.5, 93.2]
6	94.6 [93.8, 95.4]	95.8 [94.6, 96.8]	93.6 [92.4, 94.8]
7	95.7 [94.9, 96.4]	96.4 [95.3, 97.4]	95.1 [94.0, 96.1]
8	96.7 [96.0, 97.2]	97.0 [96.1, 97.9]	96.4 [95.5, 97.3]
9	97.5 [97.0, 98.0]	97.7 [96.9, 98.4]	97.5 [96.7, 98.1]
10	98.3 [97.8, 98.7]	98.4 [97.6, 98.9]	98.3 [97.6, 98.8]
11	98.8 [98.4, 99.1]	98.9 [98.2, 99.4]	98.9 [98.2, 99.3]
12	99.2 [98.8, 99.4]	99.2 [98.7, 99.6]	99.2 [98.7, 99.6]
13	99.4 [99.1, 99.6]	99.5 [99.0, 99.8]	99.3 [98.9, 99.7]
14	99.5 [99.3, 99.8]	99.6 [99.3, 99.9]	99.4 [99.0, 99.8]
15	99.7 [99.4, 99.8]	99.7 [99.4, >99.9]	99.6 [99.3, 99.9]
16	99.7 [99.6, 99.9]	99.8 [99.5, >99.9]	99.7 [99.4, 99.9]
17	99.8 [99.7, 99.9]	99.9 [99.6, >99.9]	99.8 [99.6, 99.9]
18	99.9 [99.7, >99.9]	99.9 [99.6, >99.9]	99.9 [99.7, >99.9]
19	99.9 [99.8, >99.9]	99.9 [99.7, >99.9]	99.9 [99.8, >99.9]
20	99.9 [99.9, >99.9]	99.9 [99.7, >99.9]	>99.9 [99.9, >99.9]
21	>99.9 [99.9, >99.9]	99.9 [99.8, >99.9]	>99.9 [99.9, >99.9]
22	>99.9 [99.9, >99.9]	>99.9 [99.8, >99.9]	>99.9 [>99.9, >99.9]
23	>99.9 [99.9, >99.9]	>99.9 [99.8, >99.9]	>99.9 [>99.9, >99.9]
24	>99.9 [>99.9, >99.9]	>99.9 [99.9, >99.9]	>99.9 [>99.9, >99.9]

Percentile bootstrap CIs (B = 1,000). Age-stratified norms are provided in **Supplementary Tables 8–S15**.

### 3.7 Cross-cohort comparison

Cross-cohort comparisons with the 2009 normative sample [ (34); *N* = 2,473 after age filtering to ≥ 16] are presented in **Supplementary Table 16**. At the sum score level, the GSI did not differ significantly between cohorts [*M* = 4.69, *SD* = 7.42 in 2009 vs. *M* = 4.35, *SD* = 7.62 in 2020/21; *d* = -0.05, 95% CI (-0.10, 0.01), *p* = 0.105]. SOM scores were significantly lower in the present sample [*M* = 1.48 vs. 1.21; *d* = -0.11, 95% CI (-0.16, -0.05), *p* < 0.001]. ANX scores were also lower [*M* = 1.44 vs. 1.25; *d* = -0.07, 95% CI (-0.13, -0.02), *p* = 0.006]. DEP scores did not differ significantly [*M* = 1.77 vs. 1.89; *d* = 0.04, 95% CI (-0.02, 0.09), *p* = 0.200].

At the item level, 7 of 18 items showed significantly lower scores in 2020/21. The largest decreases were observed for item 6 [feeling tense/keyed up; δ = -0.07, 95% CI (-0.09, -0.04)], item 4 [pains in heart/chest; δ = -0.05, 95% CI (-0.07, -0.03)], and item 10 [trouble getting breath; δ = -0.05, 95% CI (-0.06, -0.03)]. Items 7 (nausea/upset stomach; δ = -0.03) and 9 (suddenly scared; δ = -0.04) also showed significant decreases. Only item 14 (feeling hopeless about the future) showed a significant increase [δ = 0.04, 95% CI (0.02, 0.07), *p* = 0.006], while the remaining 10 items did not change significantly. Internal consistency was comparable across cohorts (Cronbach’s alpha GSI: 0.923 in 2009 vs. 0.932 in 2020/21). The decrease in SOM was present in both men (*d* = -0.13) and women (*d* = -0.09).

## 4 Discussion

The present CFA results provide strong support for the theorized three-factor structure of the BSI-18. The correlated three-factor model showed good fit, and all standardized factor loadings exceeded 0.70. These findings are consistent with the majority of international studies (10, 14, 16, 17, 19) and confirm that the poor fit reported by Franke et al. [ (34); CFI = 0.55] was attributable to the ADF estimator (48, 53). The high factor correlations (*r* = 0.824 to 0.913), consistent with previous research (54, 55), support the use of the GSI as a global distress index. The higher-order model showed only marginally reduced fit (Δ-CFI = 0.003), with all three factors loading highly on the general distress factor (0.920 to 0.961), suggesting that the BSI-18 can be used both at the subscale level and as a global measure.

Internal consistency was good to very good and consistent with previous findings (34, 53). The SOM subscale showed the lowest reliability (omega = 0.855), a pattern also observed in previous general population studies (34, 54). The discrepancy between omega and alpha, particularly for the GSI (0.966 vs. 0.932), reflects the unequal factor loadings and confirms the recommendation to report both coefficients (44).

Strict measurement invariance was established across gender, age, and clinical screening status (PHQ-9 ≥ 10; GAD-7 ≥ 10). The full invariance across gender extends the findings of Petrowski et al. (53) from the elderly subsample to the full adult age range. The invariance across clinical screening status indicates that the BSI-18 measures the same constructs in individuals with and without clinically relevant symptom levels, supporting its use across the full severity spectrum.

The convergent validity pattern was confirmed for depression and anxiety: the BSI-18 depression factor correlated most highly with the PHQ-9 (r = 0.912) and the anxiety factor with the GAD-7 (r = 0.889), consistent with Spitzer et al. (54). For somatization, the pattern was less clear-cut: SOM correlated more strongly with the PHQ-9 (r = 0.799) than with the CFQ-11 (r = 0.580), reflecting both the strong general distress component shared by all BSI-18 dimensions and the only partial overlap between fatigue and the broader somatization construct. The moderate SOM-CFQ-11 correlation is expected given that the CFQ-11 focuses on fatigue and does not directly assess all BSI-18 SOM symptom domains (e.g. cardiovascular, gastrointestinal complaints). The depression and anxiety factors were highly correlated (r = 0.913), and the heterotrait-monotrait ratio for this pair (HTMT = 0.919; **Supplementary Table 17**) exceeded conventional cutoffs [0.85-0.90 (56);], indicating limited discriminant validity between the two dimensions at the latent level. This is consistent with the well-documented comorbidity of depression and anxiety and with the strong general-distress component shared across the BSI-18. The superior fit of the correlated three-factor model over a unidimensional model shows that the instrument is multidimensional. However, the high depression-anxiety overlap indicates that these two subscales are not sharply separable in the present data. We therefore recommend interpreting the Global Severity Index as the primary index of overall distress, with the subscale scores providing complementary profile information. Differences between depression and anxiety subscale scores in particular should be interpreted with caution.

The availability of current population norms is essential for the clinical interpretation of screening instruments. Symptom levels in the general population are not static but change over time due to demographic shifts, changing health behaviors, and societal stressors (30, 32). From a practical perspective, these contemporary norms are indispensable for evaluating treatment efficacy: they provide the statistical basis to calculate clinical significance according to the criteria established by Jacobson and Truax (57) and to define thresholds for remission. Without updated means and standard deviations, clinicians may miscalculate whether a patient has reliably improved or successfully returned to the functional range of the current general population.

The updated norms reflect slightly lower somatization levels compared to Franke et al. (34), consistent with the cross-cohort comparison (*d* = -0.11 for SOM). The cross-cohort comparison revealed no significant increase in overall distress despite the concurrent pandemic (GSI: *d* = -0.05). This pattern is consistent with the PHQ-9 (30) and GAD-7 (32) findings using the same dataset. Notably, these findings diverge from those of the SSS-8 (33), where a substantial increase in somatic symptom burden was observed between 2012 and 2021 (*d* = -0.40). The discrepancy may reflect the different time intervals (9 years for the SSS-8 vs. 11 years for the BSI-18) and the different constructs assessed: the SSS-8 captures somatic symptom burden narrowly, whereas the BSI-18 assesses broader psychological distress across somatization, depression, and anxiety. The fact that somatic symptoms increased while broader distress remained stable suggests that the pandemic may have selectively affected physical symptom reporting rather than psychological distress in general.

The discrepancy with higher symptom levels reported in online surveys during the pandemic is likely attributable to self-selection bias in convenience samples (30, 32). The only item showing a significant increase was item 14 (feeling hopeless about the future; δ = 0.04), which may reflect a specific psychological response to pandemic-related uncertainty. Measurement invariance across the 2009 and 2020/21 cohorts was supported up to the strict level, indicating that the observed differences reflect changes in symptom reporting rather than in the psychometric functioning of the items. Two complementary mechanisms may contribute to the lower somatic symptom reporting: a recalibration of symptom appraisal through habituation to symptom-related information during the pandemic, and an attribution shift by which respondents may not have considered COVID-related somatic sensations relevant to a mental health questionnaire. The item-level pattern is characterized by decreases concentrated in somatic-autonomic items and by an isolated increase in hopelessness. This pattern is compatible with such symptom-specific processes rather than with a general decline in distress. These mechanistic interpretations remain speculative and cannot be adjudicated with cross-sectional data. Whether the present findings represent a transient shift or a new baseline remains a question for future research. The present norms thus constitute the most current representative reference for the German general population. The absence of an overall distress elevation during data collection (GSI: d = -0.05), consistent with the PHQ-9 (30) and GAD-7 (32) analyses of the same sample, suggests that they are not systematically distorted by the pandemic context.

Women reported higher scores than men on all BSI-18 scales, consistent with the broader literature on gender differences in psychological distress. The effect sizes (*d* = -0.11 to -0.14) were small but comparable to gender effects reported for the SSS-8 (*d* = -0.22; 33) and the PHQ-9 [*d* = -0.17 (30);]. The largest item-level gender difference was observed for loneliness (item 5, δ = -0.10), a depression item, rather than for somatization, which is noteworthy given the more pronounced gender differences typically observed in somatic symptom reporting. Somatization items showed only small gender effects (δ = -0.01 to -0.05), whereas cardiovascular symptoms (pains in heart/chest, trouble getting breath) did not differ significantly between genders. These item-level patterns provide a more nuanced picture than subscale-level comparisons and may inform gender-sensitive screening approaches.

### 4.1 Limitations

Despite the high-quality data from a large representative sample, this study is not without limitations. First, the response rate was 43.1%, which is comparable to similar surveys (58–60) but may still introduce non-response bias. A systematic evaluation of non-response bias is not feasible due to the lack of demographic information on non-responders. Second, no clinical interviews were conducted, limiting conclusions regarding diagnostic efficiency. Third, the cross-sectional design does not permit causal conclusions. Fourth, data analysis was based on a single imputed dataset. Fifth, data were collected during the COVID-19 pandemic, which may have influenced symptom levels. Sixth, the CFQ-11 does not assess all symptom domains covered by the BSI-18 SOM subscale, and a broad somatic symptom measure such as the PHQ-15 was not available in the survey. The convergent validity findings for SOM should therefore be interpreted accordingly. Seventh, the cross-sectional design precludes the assessment of test-retest reliability. Eighth, measurement invariance across age was examined using a median split. This maximizes subgroup sizes and thus estimability but cannot detect non-invariance confined to narrower developmental stages (e.g., adolescence or old age), and age-related heterogeneity within the two broad groups may remain undetected.

### 4.2 Conclusion

The German BSI-18 demonstrated good psychometric properties in a large representative sample. The correlated three-factor structure was confirmed using the WLSMV estimator, resolving the poor fit indices reported by Franke et al. (34). Strict measurement invariance was established across gender, age, and clinical screening status. The updated population norms and cross-cohort comparison provide a useful reference for clinical practice and epidemiological research.

## Data Availability

The datasets presented in this article are not readily available because ethics board approval did not include open data sharing. Requests to access the datasets should be directed to elmar.Braehler@medizin.uni-leipzig.de.

## Footnotes

1 Measurement invariance across the interaction of gender × age could not be estimated. After the necessary response category collapse (categories 3 and 4 merged), item 1 (faintness/dizziness) had zero observations in the highest category in one subgroup, preventing threshold estimation under WLSMV with theta parameterization.
