# Supplementary material for: Psychometric properties and population norms of the Brief Symptom Inventory-18 (BSI-18) based on a representative German sample
Source: Front Psychiatry. 2026 Aug 12;17:1877721. doi: 10.3389/fpsyt.2026.1877721 (PMC13506695; doi:10.3389/fpsyt.2026.1877721)
Supplement: Supplementary file 1 [file SupplementaryFile1.docx]

# Supplementary Material

*Supplementary Table S1. Parameter Constraints for MGCFA*

| Parameters | Threshold invariance | Weak Invariance | Strong Invariance | Full Invariance |
| --- | --- | --- | --- | --- |
| Item loadings | free | equal across groups | equal across groups | equal across groups |
| Intercepts |  |  |  |  |
| Items | constrained to 0 in first group | constrained to 0 in first group | equal (0) across groups | equal (0) across groups |
| Latent | constrained to 0 in all groups | constrained to 0 in all groups | constrained to zero in first group | constrained to zero in first group |
| Thresholds | equal across groups | equal across groups | equal across groups | equal across groups |
| Variances |  |  |  |  |
| Residual | constrained to 1 in first group | constrained to 1 in first group | constrained to 1 in first group | equal (1) across groups |
| Latent | constrained to 1 in all groups | constrained to 1 in first group | constrained to 1 in first group | constrained to 1 in first group |

*Supplementary Table S2. Internal consistency (bootstrap B = 1,000).*

|  | omega | 95% CI | alpha | 95% CI |
| --- | --- | --- | --- | --- |
| Total (*N* = 2,519) |  |  |  |  |
| GSI | 0.966 | [0.961, 0.974] | 0.932 | [0.923, 0.941] |
| SOM | 0.855 | [0.837, 0.874] | 0.839 | [0.814, 0.860] |
| DEP | 0.893 | [0.881, 0.906] | 0.872 | [0.858, 0.886] |
| ANX | 0.869 | [0.852, 0.885] | 0.852 | [0.831, 0.870] |
| Male ( *n* = 1,193) |  |  |  |  |
| GSI | 0.968 | [0.961, 0.982] | 0.936 | [0.918, 0.948] |
| SOM | 0.873 | [0.846, 0.898] | 0.861 | [0.822, 0.889] |
| DEP | 0.889 | [0.868, 0.908] | 0.871 | [0.844, 0.892] |
| ANX | 0.867 | [0.840, 0.897] | 0.850 | [0.812, 0.881] |
| Female ( *n* =1,322) |  |  |  |  |
| GSI | 0.967 | [0.960, 0.978] | 0.928 | [0.917, 0.937] |
| SOM | 0.840 | [0.816, 0.862] | 0.818 | [0.786, 0.843] |
| DEP | 0.897 | [0.882, 0.912] | 0.871 | [0.852, 0.888] |
| ANX | 0.869 | [0.850, 0.888] | 0.851 | [0.826, 0.871] |

*Note. omega via the semTools package. 95% CI = percentile bootstrap.*

*Supplementary Table S3. CFA fit indices (N = 2,519).*

| Model | chi2 | df | CFI | TLI | RMSEA | SRMR |
| --- | --- | --- | --- | --- | --- | --- |
| M1: Unidimensional | 1525 | 135 | 0.961 | 0.955 | 0.064 | 0.054 |
| M2: 3-factor correlated | 644 | 132 | 0.986 | 0.983 | 0.039 | 0.034 |
| M3: Higher-order (equal) | 732 | 134 | 0.983 | 0.981 | 0.042 | 0.039 |

*Note. WLSMV, robust (scaled) fit indices.*

*Supplementary Table S4. Standardized factor loadings (95% CI) for the unidimensional model (M1), the correlated three-factor model (M2), and the higher-order model with equality-constrained second-order loadings (M3)*

| Item | M1: GSI | M2: Factor | M2: Loading | M3: Factor | M3: Loading |
| --- | --- | --- | --- | --- | --- |
| BSI-1 | .799 [.766, .831] | SOM | .850 [.818, .882] | SOM | .886 [.864, .909] |
| BSI-4 | .806 [.775, .836] | SOM | .850 [.822, .879] | SOM | .845 [.815, .874] |
| BSI-7 | .683 [.645, .722] | SOM | .727 [.689, .766] | SOM | .721 [.682, .760] |
| BSI-10 | .809 [.778, .840] | SOM | .850 [.821, .880] | SOM | .844 [.814, .874] |
| BSI-13 | .755 [.720, .789] | SOM | .797 [.764, .830] | SOM | .790 [.757, .823] |
| BSI-16 | .827 [.803, .851] | SOM | .882 [.860, .904] | SOM | .872 [.850, .895] |
| BSI-2 | .835 [.814, .855] | DEP | .863 [.843, .883] | DEP | .873 [.856, .891] |
| BSI-5 | .795 [.770, .820] | DEP | .819 [.795, .843] | DEP | .816 [.792, .840] |
| BSI-8 | .869 [.850, .889] | DEP | .898 [.879, .916] | DEP | .895 [.877, .914] |
| BSI-11 | .876 [.856, .897] | DEP | .905 [.886, .925] | DEP | .903 [.884, .923] |
| BSI-14 | .820 [.798, .842] | DEP | .849 [.827, .870] | DEP | .846 [.824, .868] |
| BSI-17 | .825 [.782, .867] | DEP | .861 [.817, .904] | DEP | .858 [.815, .901] |
| BSI-3 | .861 [.840, .882] | ANX | .884 [.863, .905] | ANX | .849 [.830, .868] |
| BSI-6 | .745 [.717, .773] | ANX | .764 [.736, .792] | ANX | .772 [.744, .800] |
| BSI-9 | .846 [.816, .876] | ANX | .867 [.837, .896] | ANX | .875 [.845, .905] |
| BSI-12 | .896 [.870, .922] | ANX | .916 [.891, .941] | ANX | .925 [.899, .950] |
| BSI-15 | .799 [.769, .828] | ANX | .821 [.792, .850] | ANX | .829 [.800, .858] |
| BSI-18 | .859 [.835, .883] | ANX | .880 [.857, .904] | ANX | .890 [.866, .913] |

*Note. Standardized estimates (std.all) from WLSMV estimation, with 95% CI. SOM = somatization; DEP = depression; ANX = anxiety; GSI = Global Severity Index. In M3, the unstandardized second-order loadings were constrained to equality to prevent a boundary estimate (the standardized second-order loading of the anxiety factor approached unity in the unconstrained solution). Standardized values therefore differ slightly across factors. Item order follows factor assignment (somatization: items 1, 4, 7, 10, 13, 16; depression: items 2, 5, 8, 11, 14, 17; anxiety: items 3, 6, 9, 12, 15, 18).*

*Panel B. Latent factor correlations (M2)*

|  | r | 95% CI |
| --- | --- | --- |
| SOM–DEP | .824 | [.797, .850] |
| SOM–ANX | .895 | [.874, .916] |
| DEP–ANX | .913 | [.895, .931] |

*Panel C. Second-order loadings (M3, equality-constrained)*

|  | Loading | 95% CI |
| --- | --- | --- |
| GSI → SOM | .920 | [.902, .938] |
| GSI → DEP | .934 | [.919, .948] |
| GSI → ANX | .961 | [.948, .973] |

*Supplementary Table S5. BSI-18 scores by gender (N = 2,515).*

| Nr | Item | M (Men) | SD | Skew | Kurt | M (Women) | SD | Skew | Kurt | p | delta | 95% CI |
| --- | --- | --- | --- | --- | --- | --- | --- | --- | --- | --- | --- | --- |
| 1 | Faintness/dizziness | 0.12 | 0.43 | 4.68 | 26.12 | 0.19 | 0.51 | 3.22 | 11.67 | < 0.001 | -0.05 | [-0.08, -0.03] |
| 2 | No interest in things | 0.34 | 0.71 | 2.48 | 6.59 | 0.41 | 0.75 | 2.14 | 4.8 | 0.015 | -0.05 | [-0.09, -0.02] |
| 3 | Nervousness/shakiness | 0.2 | 0.57 | 3.42 | 13.37 | 0.29 | 0.62 | 2.59 | 7.57 | 0.001 | -0.07 | [-0.1, -0.04] |
| 4 | Pains in heart/chest | 0.16 | 0.48 | 3.66 | 15.63 | 0.16 | 0.48 | 3.45 | 13.29 | 0.933 | 0.00 | [-0.02, 0.03] |
| 5 | Feeling lonely | 0.36 | 0.79 | 2.57 | 6.61 | 0.54 | 0.93 | 1.85 | 2.83 | < 0.001 | -0.1 | [-0.13, -0.06] |
| 6 | Feeling tense/keyed up | 0.36 | 0.69 | 2.09 | 4.33 | 0.45 | 0.76 | 1.85 | 3.4 | 0.002 | -0.06 | [-0.09, -0.02] |
| 7 | Nausea/upset stomach | 0.19 | 0.52 | 3.43 | 14.68 | 0.27 | 0.64 | 2.86 | 9.12 | < 0.001 | -0.05 | [-0.07, -0.02] |
| 8 | Feeling blue | 0.25 | 0.65 | 3.17 | 11.03 | 0.32 | 0.71 | 2.56 | 6.72 | 0.009 | -0.05 | [-0.08, -0.02] |
| 9 | Suddenly scared | 0.09 | 0.39 | 5.58 | 37.06 | 0.14 | 0.46 | 4 | 18.07 | 0.003 | -0.04 | [-0.06, -0.02] |
| 10 | Trouble getting breath | 0.15 | 0.52 | 4.2 | 19.89 | 0.18 | 0.57 | 3.6 | 13.56 | 0.213 | -0.01 | [-0.04, 0.01] |
| 11 | Feelings of worthlessness | 0.23 | 0.67 | 3.45 | 12.5 | 0.26 | 0.68 | 3.06 | 9.68 | 0.247 | -0.02 | [-0.05, 0] |
| 12 | Spells of terror/panic | 0.08 | 0.36 | 5.55 | 36.21 | 0.12 | 0.45 | 4.57 | 24.19 | 0.011 | -0.03 | [-0.05, -0.01] |
| 13 | Numbness/tingling | 0.18 | 0.56 | 3.98 | 18.33 | 0.22 | 0.6 | 3.36 | 12.34 | 0.111 | -0.02 | [-0.05, 0] |
| 14 | Feeling hopeless | 0.41 | 0.86 | 2.39 | 5.49 | 0.5 | 0.89 | 1.96 | 3.38 | 0.015 | -0.06 | [-0.09, -0.02] |
| 15 | Feeling restless | 0.22 | 0.62 | 3.58 | 14.31 | 0.22 | 0.62 | 3.35 | 12.4 | 0.801 | -0.01 | [-0.03, 0.02] |
| 16 | Feeling weak | 0.26 | 0.66 | 3.07 | 10.15 | 0.31 | 0.71 | 2.61 | 6.91 | 0.059 | -0.03 | [-0.06, 0] |
| 17 | Thoughts of ending life | 0.06 | 0.34 | 7.23 | 60.1 | 0.06 | 0.33 | 6.76 | 53.13 | 0.907 | 0.00 | [-0.02, 0.01] |
| 18 | Feeling fearful | 0.12 | 0.44 | 4.39 | 22.03 | 0.19 | 0.53 | 3.18 | 10.79 | 0.001 | -0.05 | [-0.07, -0.02] |

*Note. Men: n = 1,193; Women: n = 1,322. Skew=skewness; Kurt=excess kurtosis. Negative=higher in women*

*Supplementary Table S6. Latent correlations between BSI-18 factors and criterion measures (WLSMV, N = 2,519).*

| BSI-18 | PHQ-9 [95% CI] | GAD-7 [95% CI] | CFQ-11 [95% CI] |
| --- | --- | --- | --- |
| GSI | 0.899 [0.879, 0.919] | 0.873 [0.853, 0.893] | 0.589 [0.546, 0.635] |
| SOM | 0.799 [0.764, 0.835] | 0.734 [0.699, 0.768] | 0.580 [0.533, 0.630] |
| DEP | 0.912 [0.893, 0.932] | 0.862 [0.839, 0.885] | 0.592 [0.547, 0.637] |
| ANX | 0.854 [0.824, 0.884] | 0.889 [0.867, 0.911] | 0.522 [0.461, 0.582] |

*Note. WLSMV estimation. Convergent pairs: DEP–PHQ-9, ANX–GAD-7, SOM–CFQ-11. 95% CI = percentile bootstrap (B = 1,000).*

*Supplementary Table S7. Measurement invariance (MGCFA, M2).*

| Comparison | Step | χ² | df | CFI | RMSEA | Delta-CFI | Delta-RMSEA | Decision |
| --- | --- | --- | --- | --- | --- | --- | --- | --- |
| Gender |  |  |  |  |  |  |  |  |
|  | Configural | 693.0 | 264 | 0.988 | 0.036 |  |  | Baseline |
|  | Threshold | 711.7 | 282 | 0.988 | 0.035 | 0.000 | -0.001 | Invariant |
|  | Weak | 700.9 | 297 | 0.989 | 0.033 | 0.001 | -0.002 | Invariant |
|  | Strong | 660.5 | 312 | 0.990 | 0.030 | 0.001 | -0.003 | Invariant |
|  | Full | 637.4 | 330 | 0.991 | 0.027 | 0.001 | -0.003 | Invariant |
| Age (median split) |  |  |  |  |  |  |  |  |
|  | Configural | 724.6 | 264 | 0.987 | 0.037 |  |  | Baseline |
|  | Threshold | 743.6 | 282 | 0.987 | 0.036 | 0.000 | -0.001 | Invariant |
|  | Weak | 748.2 | 297 | 0.988 | 0.035 | 0.001 | -0.001 | Invariant |
|  | Strong | 738.1 | 312 | 0.988 | 0.033 | 0.000 | -0.002 | Invariant |
|  | Full | 756.9 | 330 | 0.988 | 0.032 | 0.000 | -0.001 | Invariant |
| Depression (PHQ-9 >= 10) |  |  |  |  |  |  |  |  |
|  | Configural | 692.2 | 264 | 0.976 | 0.036 |  |  | Baseline |
|  | Threshold | 726.3 | 282 | 0.975 | 0.035 | -0.001 | -0.001 | Invariant |
|  | Weak | 713.4 | 297 | 0.977 | 0.033 | 0.002 | -0.002 | Invariant |
|  | Strong | 711.9 | 312 | 0.978 | 0.032 | 0.001 | -0.001 | Invariant |
|  | Full | 711.2 | 330 | 0.979 | 0.030 | 0.001 | -0.002 | Invariant |
| Anxiety (GAD-7 >= 10) |  |  |  |  |  |  |  |  |
|  | Configural | 664.9 | 264 | 0.982 | 0.035 |  |  | Baseline |
|  | Threshold | 691.0 | 282 | 0.981 | 0.034 | -0.001 | -0.001 | Invariant |
|  | Weak | 676.7 | 297 | 0.983 | 0.032 | 0.002 | -0.002 | Invariant |
|  | Strong | 685.7 | 312 | 0.983 | 0.031 | 0.000 | -0.001 | Invariant |
|  | Full | 713.7 | 330 | 0.983 | 0.030 | 0.000 | -0.001 | Invariant |

*Note. WLSMV, theta parameterization (Wu & Estabrook, 2016). Criteria: Delta-CFI >= -0.010, Delta-RMSEA < 0.015 (Chen, 2007). Gender × age could not be tested (see footnote 1 in main manuscript).*

*Supplementary Table S8. GSI norms - Men by age group.*

| Score | 16-24 [95% CI] | 25-34 [95% CI] | 35-44 [95% CI] | 45-54 [95% CI] | 55-64 [95% CI] | 65-74 [95% CI] | 75+ [95% CI] |
| --- | --- | --- | --- | --- | --- | --- | --- |
| 0 | 53.6 [43.5, 63.3] | 63.6 [56.9, 70.6] | 55.2 [48.2, 63.2] | 45.7 [38.7, 53.1] | 42.4 [35.9, 48.9] | 35.2 [28.1, 42.6] | 24.3 [14.8, 33.4] |
| 1 | 60.4 [51.3, 69.4] | 71.2 [65.7, 76.8] | 65.2 [59.4, 71.4] | 54.4 [48.2, 60.8] | 52.8 [47.3, 58.4] | 46.3 [40.1, 52.4] | 37.9 [29.6, 46.1] |
| 2 | 66.4 [58.2, 75.1] | 77.4 [72.2, 82.3] | 73.3 [68.0, 78.3] | 61.8 [56.0, 67.7] | 61.3 [56.1, 66.6] | 55.6 [49.4, 61.3] | 49.1 [41.0, 56.9] |
| 3 | 71.7 [64.2, 80.2] | 82.3 [77.5, 86.7] | 79.6 [74.7, 84.1] | 68.1 [62.2, 73.8] | 68.1 [62.8, 73.1] | 63.3 [56.8, 69.0] | 58.2 [50.0, 65.9] |
| 4 | 76.4 [68.9, 84.6] | 86.2 [81.4, 90.2] | 84.4 [79.7, 88.5] | 73.2 [67.4, 78.8] | 73.5 [67.9, 78.5] | 69.6 [62.9, 75.2] | 65.5 [57.0, 72.9] |
| 5 | 80.5 [73.6, 88.2] | 89.1 [84.6, 93.0] | 88.0 [83.3, 91.9] | 77.5 [71.8, 82.9] | 77.6 [72.2, 82.7] | 74.6 [68.1, 80.4] | 71.1 [62.5, 78.5] |
| 6 | 84.1 [77.7, 91.1] | 91.3 [87.1, 95.0] | 90.5 [86.0, 94.3] | 80.9 [75.6, 86.0] | 80.6 [75.3, 85.6] | 78.5 [72.5, 84.2] | 75.4 [66.9, 82.7] |
| 7 | 87.1 [81.1, 93.3] | 92.8 [88.9, 96.3] | 92.1 [87.9, 95.9] | 83.7 [78.8, 88.3] | 82.8 [77.6, 87.6] | 81.6 [76.0, 86.8] | 78.6 [70.6, 85.6] |
| 8 | 89.7 [84.5, 94.9] | 93.8 [90.2, 97.0] | 93.1 [89.1, 96.8] | 85.9 [81.3, 89.9] | 84.3 [79.5, 88.9] | 83.9 [78.5, 88.7] | 80.8 [72.9, 87.8] |
| 9 | 91.9 [87.4, 96.0] | 94.4 [91.0, 97.4] | 93.6 [90.0, 97.2] | 87.6 [83.3, 91.4] | 85.4 [80.6, 89.9] | 85.7 [80.5, 90.2] | 82.5 [74.7, 89.4] |
| 10 | 93.7 [89.7, 96.9] | 94.9 [91.8, 97.7] | 94.0 [90.6, 97.5] | 89.1 [84.9, 92.7] | 86.2 [81.5, 90.6] | 87.1 [82.2, 91.4] | 83.8 [76.1, 90.4] |
| 11 | 95.2 [91.6, 97.6] | 95.3 [92.3, 97.8] | 94.3 [91.0, 97.6] | 90.3 [86.2, 93.7] | 87.0 [82.4, 91.1] | 88.3 [83.7, 92.4] | 84.9 [77.3, 91.5] |
| 12 | 96.5 [93.2, 98.3] | 95.7 [92.8, 98.1] | 94.6 [91.6, 97.8] | 91.4 [87.4, 94.7] | 87.8 [83.4, 91.6] | 89.3 [84.9, 93.2] | 86.0 [78.6, 92.4] |
| 13 | 97.5 [94.6, 98.8] | 96.0 [93.3, 98.4] | 95.0 [92.2, 98.0] | 92.4 [88.7, 95.6] | 88.5 [84.3, 92.2] | 90.2 [85.9, 93.9] | 87.0 [79.9, 93.3] |
| 14 | 98.2 [95.6, 99.2] | 96.4 [93.8, 98.6] | 95.5 [92.6, 98.2] | 93.2 [89.6, 96.2] | 89.3 [85.3, 92.9] | 91.0 [87.0, 94.6] | 88.0 [81.2, 94.1] |
| 15 | 98.8 [96.4, 99.6] | 96.7 [94.2, 98.7] | 95.9 [93.3, 98.4] | 94.0 [90.6, 96.8] | 90.1 [86.2, 93.5] | 91.7 [87.7, 95.1] | 88.9 [82.5, 94.7] |
| 16 | 99.2 [97.1, 99.8] | 97.0 [94.7, 98.9] | 96.4 [93.8, 98.6] | 94.6 [91.3, 97.3] | 90.8 [87.1, 94.1] | 92.3 [88.5, 95.6] | 89.8 [83.8, 95.1] |
| 17 | 99.5 [97.8, >99.9] | 97.3 [95.2, 99.1] | 96.8 [94.3, 98.8] | 95.2 [92.0, 97.6] | 91.5 [88.0, 94.6] | 92.8 [89.1, 95.9] | 90.7 [84.8, 95.7] |
| 18 | 99.7 [98.2, >99.9] | 97.6 [95.5, 99.2] | 97.3 [94.8, 99.0] | 95.6 [92.5, 98.0] | 92.2 [88.7, 95.2] | 93.3 [89.6, 96.2] | 91.5 [85.9, 96.0] |
| 19 | 99.8 [98.7, >99.9] | 97.8 [95.9, 99.3] | 97.7 [95.2, 99.2] | 96.0 [93.0, 98.3] | 92.8 [89.4, 95.7] | 93.7 [90.2, 96.6] | 92.3 [86.9, 96.6] |
| 20 | 99.8 [99.0, >99.9] | 98.1 [96.2, 99.4] | 98.1 [95.7, 99.4] | 96.3 [93.4, 98.5] | 93.4 [90.0, 96.3] | 94.1 [90.6, 96.9] | 93.0 [88.0, 96.9] |
| 21 | 99.9 [99.3, >99.9] | 98.3 [96.5, 99.6] | 98.4 [96.1, 99.5] | 96.6 [93.8, 98.7] | 94.0 [90.6, 96.7] | 94.5 [91.0, 97.2] | 93.7 [89.1, 97.4] |
| 22 | 99.9 [99.5, >99.9] | 98.5 [96.7, 99.7] | 98.7 [96.5, 99.6] | 96.9 [94.1, 98.9] | 94.5 [91.2, 97.1] | 94.8 [91.5, 97.4] | 94.4 [90.1, 97.9] |
| 23 | 99.9 [99.7, >99.9] | 98.6 [97.0, 99.8] | 98.9 [96.8, 99.8] | 97.1 [94.4, 99.0] | 94.9 [91.7, 97.4] | 95.1 [91.9, 97.7] | 95.1 [91.0, 98.3] |
| 24 | 99.9 [99.8, >99.9] | 98.8 [97.2, 99.8] | 99.1 [97.2, 99.9] | 97.2 [94.7, 99.2] | 95.3 [92.3, 97.7] | 95.5 [92.3, 97.9] | 95.7 [91.9, 98.7] |
| 25 | 99.9 [99.9, >99.9] | 98.9 [97.4, 99.9] | 99.2 [97.6, 99.9] | 97.4 [95.0, 99.3] | 95.6 [92.7, 97.9] | 95.8 [92.9, 98.1] | 96.3 [92.7, 99.0] |
| 26 | >99.9 [99.9, >99.9] | 99.0 [97.6, >99.9] | 99.3 [97.9, >99.9] | 97.5 [95.1, 99.4] | 95.9 [93.2, 98.1] | 96.1 [93.3, 98.3] | 96.8 [93.6, 99.2] |
| 27 | >99.9 [99.9, >99.9] | 99.1 [97.8, >99.9] | 99.4 [98.2, >99.9] | 97.7 [95.4, 99.5] | 96.2 [93.6, 98.3] | 96.4 [93.8, 98.5] | 97.3 [94.3, 99.4] |
| 28 | >99.9 [99.9, >99.9] | 99.2 [97.9, >99.9] | 99.5 [98.4, >99.9] | 97.8 [95.6, 99.5] | 96.4 [93.9, 98.4] | 96.7 [94.1, 98.7] | 97.8 [95.0, 99.5] |
| 29 | >99.9 [99.9, >99.9] | 99.3 [98.1, >99.9] | 99.5 [98.7, >99.9] | 97.9 [95.7, 99.6] | 96.6 [94.2, 98.5] | 96.9 [94.5, 98.8] | 98.2 [95.5, 99.7] |
| 30 | >99.9 [99.9, >99.9] | 99.4 [98.2, >99.9] | 99.6 [98.9, >99.9] | 98.0 [95.9, 99.7] | 96.8 [94.5, 98.7] | 97.2 [94.9, 99.0] | 98.6 [96.1, 99.7] |
| 31 | >99.9 [>99.9, >99.9] | 99.4 [98.3, >99.9] | 99.6 [99.1, >99.9] | 98.1 [96.1, 99.7] | 97.0 [94.8, 98.8] | 97.4 [95.2, 99.1] | 98.9 [96.6, 99.8] |
| 32 | >99.9 [>99.9, >99.9] | 99.5 [98.4, >99.9] | 99.7 [99.2, >99.9] | 98.2 [96.3, 99.7] | 97.2 [95.0, 98.9] | 97.7 [95.5, 99.3] | 99.1 [97.1, 99.9] |
| 33 | >99.9 [>99.9, >99.9] | 99.6 [98.5, >99.9] | 99.7 [99.3, >99.9] | 98.4 [96.6, 99.8] | 97.3 [95.1, 99.0] | 97.9 [95.8, 99.5] | 99.3 [97.4, 99.9] |
| 34 | >99.9 [>99.9, >99.9] | 99.6 [98.7, >99.9] | 99.7 [99.4, >99.9] | 98.5 [96.9, 99.8] | 97.4 [95.3, 99.1] | 98.1 [96.0, 99.6] | 99.5 [97.8, >99.9] |
| 35 | >99.9 [>99.9, >99.9] | 99.6 [98.8, >99.9] | 99.8 [99.5, >99.9] | 98.7 [97.1, 99.9] | 97.6 [95.4, 99.2] | 98.3 [96.3, 99.7] | 99.6 [98.1, >99.9] |
| 36 | >99.9 [>99.9, >99.9] | 99.7 [98.9, >99.9] | 99.8 [99.6, >99.9] | 98.8 [97.4, 99.9] | 97.7 [95.6, 99.2] | 98.5 [96.6, 99.7] | 99.7 [98.3, >99.9] |
| 37 | >99.9 [>99.9, >99.9] | 99.7 [99.0, >99.9] | 99.8 [99.7, >99.9] | 99.0 [97.7, 99.9] | 97.8 [95.7, 99.3] | 98.6 [96.9, 99.8] | 99.8 [98.6, >99.9] |
| 38 | >99.9 [>99.9, >99.9] | 99.7 [99.2, >99.9] | 99.9 [99.7, >99.9] | 99.1 [97.9, 99.9] | 97.9 [95.8, 99.4] | 98.8 [97.1, 99.8] | 99.9 [98.9, >99.9] |
| 39 | >99.9 [>99.9, >99.9] | 99.8 [99.3, >99.9] | 99.9 [99.8, >99.9] | 99.2 [98.2, 99.9] | 98.0 [95.9, 99.5] | 98.9 [97.4, 99.9] | 99.9 [99.1, >99.9] |
| 40 | >99.9 [>99.9, >99.9] | 99.8 [99.4, >99.9] | 99.9 [99.8, >99.9] | 99.4 [98.4, >99.9] | 98.1 [96.0, 99.5] | 99.1 [97.6, 99.9] | 99.9 [99.3, >99.9] |
| 41 | >99.9 [>99.9, >99.9] | 99.8 [99.4, >99.9] | >99.9 [99.9, >99.9] | 99.5 [98.5, >99.9] | 98.1 [96.1, 99.5] | 99.2 [97.8, 99.9] | 99.9 [99.4, >99.9] |
| 42 | >99.9 [>99.9, >99.9] | 99.8 [99.5, >99.9] | >99.9 [99.9, >99.9] | 99.6 [98.7, >99.9] | 98.2 [96.2, 99.6] | 99.3 [98.0, 99.9] | 99.9 [99.6, >99.9] |
| 43 | >99.9 [>99.9, >99.9] | 99.8 [99.6, >99.9] | >99.9 [99.9, >99.9] | 99.7 [98.9, >99.9] | 98.3 [96.3, 99.6] | 99.4 [98.3, >99.9] | 99.9 [99.7, >99.9] |
| 44 | >99.9 [>99.9, >99.9] | 99.9 [99.6, >99.9] | >99.9 [99.9, >99.9] | 99.7 [99.1, >99.9] | 98.4 [96.4, 99.7] | 99.5 [98.5, >99.9] | 99.9 [99.7, >99.9] |
| 45 | >99.9 [>99.9, >99.9] | 99.9 [99.7, >99.9] | >99.9 [99.9, >99.9] | 99.8 [99.2, >99.9] | 98.4 [96.6, 99.7] | 99.5 [98.7, >99.9] | >99.9 [99.8, >99.9] |
| 46 | >99.9 [>99.9, >99.9] | 99.9 [99.7, >99.9] | >99.9 [>99.9, >99.9] | 99.8 [99.3, >99.9] | 98.5 [96.7, 99.7] | 99.6 [98.9, >99.9] | >99.9 [99.8, >99.9] |
| 47 | >99.9 [>99.9, >99.9] | 99.9 [99.8, >99.9] | >99.9 [>99.9, >99.9] | 99.9 [99.5, >99.9] | 98.6 [96.8, 99.7] | 99.7 [99.1, >99.9] | >99.9 [99.8, >99.9] |
| 48 | >99.9 [>99.9, >99.9] | 99.9 [99.8, >99.9] | >99.9 [>99.9, >99.9] | 99.9 [99.6, >99.9] | 98.6 [96.9, 99.8] | 99.7 [99.3, >99.9] | >99.9 [99.9, >99.9] |
| 49 | >99.9 [>99.9, >99.9] | 99.9 [99.8, >99.9] | >99.9 [>99.9, >99.9] | 99.9 [99.7, >99.9] | 98.7 [97.1, 99.8] | 99.8 [99.4, >99.9] | >99.9 [99.9, >99.9] |
| 50 | >99.9 [>99.9, >99.9] | 99.9 [99.9, >99.9] | >99.9 [>99.9, >99.9] | 99.9 [99.7, >99.9] | 98.8 [97.2, 99.8] | 99.8 [99.6, >99.9] | >99.9 [99.9, >99.9] |
| 51 | >99.9 [>99.9, >99.9] | >99.9 [99.9, >99.9] | >99.9 [>99.9, >99.9] | >99.9 [99.8, >99.9] | 98.8 [97.3, 99.9] | 99.8 [99.7, >99.9] | >99.9 [99.9, >99.9] |
| 52 | >99.9 [>99.9, >99.9] | >99.9 [99.9, >99.9] | >99.9 [>99.9, >99.9] | >99.9 [99.9, >99.9] | 98.9 [97.4, 99.9] | 99.9 [99.8, >99.9] | >99.9 [>99.9, >99.9] |
| 53 | >99.9 [>99.9, >99.9] | >99.9 [99.9, >99.9] | >99.9 [>99.9, >99.9] | >99.9 [99.9, >99.9] | 99.0 [97.6, 99.9] | 99.9 [99.8, >99.9] | >99.9 [>99.9, >99.9] |
| 54 | >99.9 [>99.9, >99.9] | >99.9 [99.9, >99.9] | >99.9 [>99.9, >99.9] | >99.9 [99.9, >99.9] | 99.0 [97.7, 99.9] | 99.9 [99.9, >99.9] | >99.9 [>99.9, >99.9] |
| 55 | >99.9 [>99.9, >99.9] | >99.9 [>99.9, >99.9] | >99.9 [>99.9, >99.9] | >99.9 [99.9, >99.9] | 99.1 [97.8, 99.9] | 99.9 [99.9, >99.9] | >99.9 [>99.9, >99.9] |
| 56 | >99.9 [>99.9, >99.9] | >99.9 [>99.9, >99.9] | >99.9 [>99.9, >99.9] | >99.9 [>99.9, >99.9] | 99.2 [97.9, >99.9] | >99.9 [99.9, >99.9] | >99.9 [>99.9, >99.9] |
| 57 | >99.9 [>99.9, >99.9] | >99.9 [>99.9, >99.9] | >99.9 [>99.9, >99.9] | >99.9 [>99.9, >99.9] | 99.2 [98.1, >99.9] | >99.9 [99.9, >99.9] | >99.9 [>99.9, >99.9] |
| 58 | >99.9 [>99.9, >99.9] | >99.9 [>99.9, >99.9] | >99.9 [>99.9, >99.9] | >99.9 [>99.9, >99.9] | 99.3 [98.2, >99.9] | >99.9 [>99.9, >99.9] | >99.9 [>99.9, >99.9] |
| 59 | >99.9 [>99.9, >99.9] | >99.9 [>99.9, >99.9] | >99.9 [>99.9, >99.9] | >99.9 [>99.9, >99.9] | 99.3 [98.3, >99.9] | >99.9 [>99.9, >99.9] | >99.9 [>99.9, >99.9] |
| 60 | >99.9 [>99.9, >99.9] | >99.9 [>99.9, >99.9] | >99.9 [>99.9, >99.9] | >99.9 [>99.9, >99.9] | 99.4 [98.5, >99.9] | >99.9 [>99.9, >99.9] | >99.9 [>99.9, >99.9] |
| 61 | >99.9 [>99.9, >99.9] | >99.9 [>99.9, >99.9] | >99.9 [>99.9, >99.9] | >99.9 [>99.9, >99.9] | 99.4 [98.6, >99.9] | >99.9 [>99.9, >99.9] | >99.9 [>99.9, >99.9] |
| 62 | >99.9 [>99.9, >99.9] | >99.9 [>99.9, >99.9] | >99.9 [>99.9, >99.9] | >99.9 [>99.9, >99.9] | 99.5 [98.7, >99.9] | >99.9 [>99.9, >99.9] | >99.9 [>99.9, >99.9] |
| 63 | >99.9 [>99.9, >99.9] | >99.9 [>99.9, >99.9] | >99.9 [>99.9, >99.9] | >99.9 [>99.9, >99.9] | 99.5 [98.8, >99.9] | >99.9 [>99.9, >99.9] | >99.9 [>99.9, >99.9] |
| 64 | >99.9 [>99.9, >99.9] | >99.9 [>99.9, >99.9] | >99.9 [>99.9, >99.9] | >99.9 [>99.9, >99.9] | 99.6 [98.8, >99.9] | >99.9 [>99.9, >99.9] | >99.9 [>99.9, >99.9] |
| 65 | >99.9 [>99.9, >99.9] | >99.9 [>99.9, >99.9] | >99.9 [>99.9, >99.9] | >99.9 [>99.9, >99.9] | 99.6 [98.8, >99.9] | >99.9 [>99.9, >99.9] | >99.9 [>99.9, >99.9] |
| 66 | >99.9 [>99.9, >99.9] | >99.9 [>99.9, >99.9] | >99.9 [>99.9, >99.9] | >99.9 [>99.9, >99.9] | 99.7 [98.9, >99.9] | >99.9 [>99.9, >99.9] | >99.9 [>99.9, >99.9] |
| 67 | >99.9 [>99.9, >99.9] | >99.9 [>99.9, >99.9] | >99.9 [>99.9, >99.9] | >99.9 [>99.9, >99.9] | 99.7 [98.9, >99.9] | >99.9 [>99.9, >99.9] | >99.9 [>99.9, >99.9] |
| 68 | >99.9 [>99.9, >99.9] | >99.9 [>99.9, >99.9] | >99.9 [>99.9, >99.9] | >99.9 [>99.9, >99.9] | 99.7 [99.0, >99.9] | >99.9 [>99.9, >99.9] | >99.9 [>99.9, >99.9] |
| 69 | >99.9 [>99.9, >99.9] | >99.9 [>99.9, >99.9] | >99.9 [>99.9, >99.9] | >99.9 [>99.9, >99.9] | 99.8 [99.0, >99.9] | >99.9 [>99.9, >99.9] | >99.9 [>99.9, >99.9] |
| 70 | >99.9 [>99.9, >99.9] | >99.9 [>99.9, >99.9] | >99.9 [>99.9, >99.9] | >99.9 [>99.9, >99.9] | 99.8 [99.0, >99.9] | >99.9 [>99.9, >99.9] | >99.9 [>99.9, >99.9] |
| 71 | >99.9 [>99.9, >99.9] | >99.9 [>99.9, >99.9] | >99.9 [>99.9, >99.9] | >99.9 [>99.9, >99.9] | 99.8 [99.0, >99.9] | >99.9 [>99.9, >99.9] | >99.9 [>99.9, >99.9] |
| 72 | >99.9 [>99.9, >99.9] | >99.9 [>99.9, >99.9] | >99.9 [>99.9, >99.9] | >99.9 [>99.9, >99.9] | 99.9 [99.1, >99.9] | >99.9 [>99.9, >99.9] | >99.9 [>99.9, >99.9] |

*Note. Smoothed cumulative percentiles (SCAM). 95% CI = bootstrap (B = 1,000).*

*Supplementary Table S9. GSI norms - Women by age group.*

| Score | 16-24 [95% CI] | 25-34 [95% CI] | 35-44 [95% CI] | 45-54 [95% CI] | 55-64 [95% CI] | 65-74 [95% CI] | 75+ [95% CI] |
| --- | --- | --- | --- | --- | --- | --- | --- |
| 0 | 37.1 [28.4, 46.4] | 48.0 [39.7, 55.3] | 46.3 [40.0, 52.3] | 42.2 [35.4, 49.1] | 37.4 [31.1, 44.1] | 35.3 [28.7, 42.4] | 18.1 [10.8, 25.5] |
| 1 | 47.7 [40.4, 56.1] | 55.2 [48.1, 61.8] | 55.8 [50.4, 61.4] | 53.0 [46.8, 58.6] | 48.3 [42.6, 53.8] | 45.5 [39.6, 51.5] | 29.1 [22.6, 35.9] |
| 2 | 56.6 [49.5, 64.3] | 61.4 [54.8, 68.0] | 63.7 [58.6, 68.9] | 61.9 [56.2, 67.0] | 57.2 [51.7, 62.0] | 54.0 [48.0, 59.8] | 38.6 [32.0, 45.4] |
| 3 | 63.8 [56.6, 71.0] | 66.9 [60.6, 73.2] | 70.1 [64.9, 75.3] | 69.0 [63.6, 74.0] | 64.3 [58.7, 69.0] | 61.0 [54.7, 66.8] | 46.8 [39.7, 54.2] |
| 4 | 69.6 [62.5, 76.4] | 71.6 [65.5, 77.8] | 75.1 [69.9, 80.3] | 74.7 [69.4, 79.6] | 69.8 [64.2, 74.6] | 66.6 [60.3, 72.5] | 53.8 [46.7, 61.4] |
| 5 | 74.2 [67.2, 80.9] | 75.6 [69.9, 81.6] | 79.0 [73.8, 84.1] | 79.1 [74.1, 84.1] | 74.0 [68.5, 78.7] | 71.1 [64.8, 77.1] | 59.8 [52.6, 67.4] |
| 6 | 77.7 [70.8, 84.2] | 79.1 [73.5, 84.7] | 82.0 [77.0, 86.9] | 82.4 [77.6, 87.3] | 77.0 [71.6, 81.7] | 74.6 [68.5, 80.3] | 64.8 [57.6, 72.0] |
| 7 | 80.3 [73.6, 86.6] | 81.9 [76.7, 87.3] | 84.1 [79.3, 88.7] | 84.8 [80.4, 89.5] | 79.2 [74.0, 83.9] | 77.4 [71.6, 82.8] | 69.0 [62.1, 75.8] |
| 8 | 82.3 [76.0, 88.2] | 84.3 [79.3, 89.1] | 85.6 [81.1, 90.0] | 86.6 [82.4, 91.1] | 80.7 [75.8, 85.3] | 79.6 [74.1, 84.6] | 72.5 [66.0, 78.9] |
| 9 | 83.8 [77.8, 89.8] | 86.3 [81.3, 90.8] | 86.7 [82.4, 90.9] | 87.8 [83.7, 92.2] | 81.8 [77.1, 86.2] | 81.3 [76.1, 86.4] | 75.4 [69.2, 81.4] |
| 10 | 85.1 [79.3, 90.6] | 88.0 [83.1, 91.9] | 87.6 [83.5, 91.6] | 88.8 [84.9, 92.9] | 82.7 [78.2, 87.1] | 82.9 [77.8, 87.7] | 77.8 [71.5, 83.8] |
| 11 | 86.2 [80.7, 91.6] | 89.3 [84.7, 93.0] | 88.3 [84.3, 92.2] | 89.7 [86.0, 93.5] | 83.6 [79.2, 88.0] | 84.4 [79.5, 88.9] | 79.9 [73.6, 85.7] |
| 12 | 87.3 [81.9, 92.6] | 90.4 [86.0, 94.1] | 89.0 [85.1, 92.7] | 90.6 [87.1, 94.2] | 84.6 [80.4, 88.8] | 85.9 [81.2, 90.2] | 81.7 [75.6, 87.4] |
| 13 | 88.4 [83.2, 93.4] | 91.3 [87.1, 95.0] | 89.6 [85.9, 93.3] | 91.4 [87.9, 94.9] | 85.6 [81.6, 89.8] | 87.4 [83.1, 91.5] | 83.2 [77.2, 88.8] |
| 14 | 89.4 [84.5, 94.1] | 92.1 [87.8, 95.7] | 90.2 [86.5, 93.8] | 92.2 [88.9, 95.5] | 86.7 [82.8, 90.7] | 88.9 [84.8, 92.7] | 84.6 [78.8, 90.0] |
| 15 | 90.4 [85.7, 94.9] | 92.7 [88.6, 96.2] | 90.8 [87.2, 94.3] | 93.0 [89.8, 96.1] | 87.8 [84.0, 91.5] | 90.4 [86.7, 93.7] | 85.7 [79.9, 91.0] |
| 16 | 91.4 [86.9, 95.5] | 93.1 [89.3, 96.6] | 91.4 [87.9, 94.8] | 93.7 [90.6, 96.5] | 88.9 [85.3, 92.5] | 91.8 [88.5, 94.7] | 86.7 [80.8, 91.8] |
| 17 | 92.3 [88.1, 96.1] | 93.5 [89.7, 97.0] | 91.9 [88.5, 95.2] | 94.4 [91.4, 97.0] | 90.0 [86.5, 93.4] | 93.1 [90.2, 95.5] | 87.6 [81.8, 92.6] |
| 18 | 93.2 [89.1, 96.6] | 93.8 [90.2, 97.3] | 92.4 [89.0, 95.5] | 95.0 [92.1, 97.3] | 91.1 [87.7, 94.2] | 94.3 [91.8, 96.4] | 88.3 [82.6, 93.3] |
| 19 | 94.0 [90.0, 97.2] | 94.1 [90.6, 97.5] | 92.8 [89.6, 95.9] | 95.6 [92.9, 97.7] | 92.1 [88.8, 95.1] | 95.4 [93.0, 97.2] | 89.1 [83.6, 93.9] |
| 20 | 94.7 [90.9, 97.6] | 94.4 [91.1, 97.6] | 93.2 [90.0, 96.3] | 96.1 [93.4, 98.1] | 93.0 [89.9, 95.9] | 96.4 [94.3, 97.8] | 89.8 [84.4, 94.5] |
| 21 | 95.4 [91.8, 98.1] | 94.7 [91.6, 97.8] | 93.7 [90.5, 96.7] | 96.5 [93.9, 98.4] | 93.9 [90.8, 96.6] | 97.2 [95.3, 98.3] | 90.5 [85.4, 95.0] |
| 22 | 96.0 [92.7, 98.5] | 95.1 [92.1, 98.0] | 94.0 [90.9, 96.9] | 96.8 [94.4, 98.7] | 94.6 [91.7, 97.2] | 97.9 [96.1, 98.8] | 91.2 [86.3, 95.6] |
| 23 | 96.6 [93.5, 98.9] | 95.5 [92.7, 98.2] | 94.4 [91.3, 97.2] | 97.1 [94.7, 99.0] | 95.3 [92.5, 97.8] | 98.4 [96.7, 99.2] | 92.0 [87.3, 96.1] |
| 24 | 97.1 [94.2, 99.2] | 95.9 [93.3, 98.4] | 94.7 [91.8, 97.4] | 97.3 [95.0, 99.2] | 95.8 [93.2, 98.2] | 98.8 [97.2, 99.5] | 92.7 [88.3, 96.6] |
| 25 | 97.6 [95.0, 99.5] | 96.4 [94.0, 98.6] | 95.1 [92.1, 97.6] | 97.5 [95.2, 99.3] | 96.3 [93.9, 98.5] | 99.1 [97.5, 99.7] | 93.5 [89.4, 97.0] |
| 26 | 98.0 [95.7, 99.7] | 96.8 [94.6, 98.8] | 95.4 [92.5, 97.8] | 97.6 [95.4, 99.4] | 96.8 [94.5, 98.8] | 99.4 [97.8, 99.9] | 94.2 [90.5, 97.4] |
| 27 | 98.4 [96.4, 99.9] | 97.3 [95.3, 99.0] | 95.7 [92.8, 98.0] | 97.7 [95.6, 99.5] | 97.1 [95.1, 99.0] | 99.5 [97.9, >99.9] | 94.9 [91.6, 97.8] |
| 28 | 98.7 [97.0, 99.9] | 97.7 [95.8, 99.2] | 95.9 [93.2, 98.2] | 97.8 [95.8, 99.6] | 97.4 [95.6, 99.1] | 99.6 [98.1, >99.9] | 95.6 [92.6, 98.1] |
| 29 | 99.0 [97.6, >99.9] | 98.1 [96.3, 99.3] | 96.2 [93.5, 98.3] | 97.9 [96.0, 99.7] | 97.7 [96.1, 99.3] | 99.6 [98.2, >99.9] | 96.3 [93.5, 98.4] |
| 30 | 99.2 [98.1, >99.9] | 98.4 [96.7, 99.5] | 96.4 [93.8, 98.4] | 98.0 [96.2, 99.7] | 98.0 [96.4, 99.4] | 99.7 [98.3, >99.9] | 96.9 [94.4, 98.7] |
| 31 | 99.4 [98.6, >99.9] | 98.8 [97.0, 99.6] | 96.6 [94.1, 98.5] | 98.1 [96.4, 99.7] | 98.2 [96.7, 99.5] | 99.7 [98.3, >99.9] | 97.4 [95.1, 99.0] |
| 32 | 99.6 [98.9, >99.9] | 99.0 [97.4, 99.7] | 96.9 [94.4, 98.7] | 98.2 [96.6, 99.8] | 98.4 [97.0, 99.6] | 99.7 [98.4, >99.9] | 97.9 [95.8, 99.3] |
| 33 | 99.7 [99.1, >99.9] | 99.3 [97.7, 99.8] | 97.1 [94.7, 98.8] | 98.4 [96.9, 99.8] | 98.6 [97.3, 99.7] | 99.7 [98.5, >99.9] | 98.3 [96.4, 99.5] |
| 34 | 99.8 [99.3, >99.9] | 99.4 [98.0, 99.9] | 97.3 [94.9, 98.9] | 98.5 [97.2, 99.9] | 98.8 [97.6, 99.8] | 99.7 [98.6, >99.9] | 98.6 [96.9, 99.7] |
| 35 | 99.9 [99.4, >99.9] | 99.6 [98.3, >99.9] | 97.5 [95.3, 99.0] | 98.7 [97.5, 99.9] | 99.0 [97.8, 99.9] | 99.7 [98.7, >99.9] | 98.9 [97.4, 99.8] |
| 36 | 99.9 [99.5, >99.9] | 99.7 [98.6, >99.9] | 97.6 [95.6, 99.1] | 98.9 [97.8, 99.9] | 99.2 [98.1, >99.9] | 99.7 [98.8, >99.9] | 99.1 [97.9, 99.9] |
| 37 | >99.9 [99.6, >99.9] | 99.7 [98.8, >99.9] | 97.8 [95.9, 99.2] | 99.0 [98.1, 99.9] | 99.3 [98.4, >99.9] | 99.7 [98.9, >99.9] | 99.3 [98.3, >99.9] |
| 38 | >99.9 [99.7, >99.9] | 99.8 [99.0, >99.9] | 98.0 [96.1, 99.3] | 99.2 [98.4, 99.9] | 99.4 [98.6, >99.9] | 99.7 [99.0, >99.9] | 99.5 [98.6, >99.9] |
| 39 | >99.9 [99.7, >99.9] | 99.8 [99.2, >99.9] | 98.1 [96.3, 99.4] | 99.4 [98.7, 99.9] | 99.6 [98.9, >99.9] | 99.7 [99.1, >99.9] | 99.6 [98.9, >99.9] |
| 40 | >99.9 [99.8, >99.9] | 99.9 [99.3, >99.9] | 98.2 [96.5, 99.5] | 99.5 [99.0, >99.9] | 99.7 [99.1, >99.9] | 99.7 [99.2, >99.9] | 99.7 [99.2, >99.9] |
| 41 | >99.9 [99.8, >99.9] | 99.9 [99.5, >99.9] | 98.4 [96.7, 99.6] | 99.6 [99.2, >99.9] | 99.7 [99.2, >99.9] | 99.7 [99.2, >99.9] | 99.8 [99.4, >99.9] |
| 42 | >99.9 [99.9, >99.9] | 99.9 [99.6, >99.9] | 98.5 [96.9, 99.7] | 99.7 [99.3, >99.9] | 99.8 [99.4, >99.9] | 99.8 [99.3, >99.9] | 99.9 [99.6, >99.9] |
| 43 | >99.9 [99.9, >99.9] | 99.9 [99.7, >99.9] | 98.6 [97.1, 99.8] | 99.8 [99.5, >99.9] | 99.9 [99.5, >99.9] | 99.8 [99.4, >99.9] | 99.9 [99.8, >99.9] |
| 44 | >99.9 [99.9, >99.9] | 99.9 [99.7, >99.9] | 98.7 [97.2, 99.9] | 99.9 [99.6, >99.9] | 99.9 [99.6, >99.9] | 99.8 [99.4, >99.9] | >99.9 [99.9, >99.9] |
| 45 | >99.9 [99.9, >99.9] | 99.9 [99.8, >99.9] | 98.8 [97.4, 99.9] | 99.9 [99.7, >99.9] | >99.9 [99.7, >99.9] | 99.8 [99.5, >99.9] | >99.9 [99.9, >99.9] |
| 46 | >99.9 [>99.9, >99.9] | 99.9 [99.8, >99.9] | 98.9 [97.6, >99.9] | >99.9 [99.7, >99.9] | >99.9 [99.7, >99.9] | 99.8 [99.6, >99.9] | >99.9 [99.9, >99.9] |
| 47 | >99.9 [>99.9, >99.9] | 99.9 [99.8, >99.9] | 99.0 [97.8, >99.9] | >99.9 [99.8, >99.9] | >99.9 [99.8, >99.9] | 99.9 [99.7, >99.9] | >99.9 [>99.9, >99.9] |
| 48 | >99.9 [>99.9, >99.9] | >99.9 [99.9, >99.9] | 99.1 [97.9, >99.9] | >99.9 [99.8, >99.9] | >99.9 [99.8, >99.9] | 99.9 [99.7, >99.9] | >99.9 [>99.9, >99.9] |
| 49 | >99.9 [>99.9, >99.9] | >99.9 [99.9, >99.9] | 99.2 [98.1, >99.9] | >99.9 [99.9, >99.9] | >99.9 [99.8, >99.9] | 99.9 [99.8, >99.9] | >99.9 [>99.9, >99.9] |
| 50 | >99.9 [>99.9, >99.9] | >99.9 [99.9, >99.9] | 99.2 [98.2, >99.9] | >99.9 [99.9, >99.9] | >99.9 [99.9, >99.9] | 99.9 [99.9, >99.9] | >99.9 [>99.9, >99.9] |
| 51 | >99.9 [>99.9, >99.9] | >99.9 [99.9, >99.9] | 99.3 [98.3, >99.9] | >99.9 [99.9, >99.9] | >99.9 [99.9, >99.9] | >99.9 [99.9, >99.9] | >99.9 [>99.9, >99.9] |
| 52 | >99.9 [>99.9, >99.9] | >99.9 [>99.9, >99.9] | 99.4 [98.5, >99.9] | >99.9 [>99.9, >99.9] | >99.9 [99.9, >99.9] | >99.9 [99.9, >99.9] | >99.9 [>99.9, >99.9] |
| 53 | >99.9 [>99.9, >99.9] | >99.9 [>99.9, >99.9] | 99.4 [98.6, >99.9] | >99.9 [>99.9, >99.9] | >99.9 [99.9, >99.9] | >99.9 [99.9, >99.9] | >99.9 [>99.9, >99.9] |
| 54 | >99.9 [>99.9, >99.9] | >99.9 [>99.9, >99.9] | 99.5 [98.8, >99.9] | >99.9 [>99.9, >99.9] | >99.9 [>99.9, >99.9] | >99.9 [>99.9, >99.9] | >99.9 [>99.9, >99.9] |
| 55 | >99.9 [>99.9, >99.9] | >99.9 [>99.9, >99.9] | 99.6 [98.9, >99.9] | >99.9 [>99.9, >99.9] | >99.9 [>99.9, >99.9] | >99.9 [>99.9, >99.9] | >99.9 [>99.9, >99.9] |
| 56 | >99.9 [>99.9, >99.9] | >99.9 [>99.9, >99.9] | 99.6 [99.1, >99.9] | >99.9 [>99.9, >99.9] | >99.9 [>99.9, >99.9] | >99.9 [>99.9, >99.9] | >99.9 [>99.9, >99.9] |
| 57 | >99.9 [>99.9, >99.9] | >99.9 [>99.9, >99.9] | 99.7 [99.2, >99.9] | >99.9 [>99.9, >99.9] | >99.9 [>99.9, >99.9] | >99.9 [>99.9, >99.9] | >99.9 [>99.9, >99.9] |
| 58 | >99.9 [>99.9, >99.9] | >99.9 [>99.9, >99.9] | 99.7 [99.4, >99.9] | >99.9 [>99.9, >99.9] | >99.9 [>99.9, >99.9] | >99.9 [>99.9, >99.9] | >99.9 [>99.9, >99.9] |
| 59 | >99.9 [>99.9, >99.9] | >99.9 [>99.9, >99.9] | 99.8 [99.5, >99.9] | >99.9 [>99.9, >99.9] | >99.9 [>99.9, >99.9] | >99.9 [>99.9, >99.9] | >99.9 [>99.9, >99.9] |
| 60 | >99.9 [>99.9, >99.9] | >99.9 [>99.9, >99.9] | 99.8 [99.6, >99.9] | >99.9 [>99.9, >99.9] | >99.9 [>99.9, >99.9] | >99.9 [>99.9, >99.9] | >99.9 [>99.9, >99.9] |
| 61 | >99.9 [>99.9, >99.9] | >99.9 [>99.9, >99.9] | 99.9 [99.7, >99.9] | >99.9 [>99.9, >99.9] | >99.9 [>99.9, >99.9] | >99.9 [>99.9, >99.9] | >99.9 [>99.9, >99.9] |
| 62 | >99.9 [>99.9, >99.9] | >99.9 [>99.9, >99.9] | 99.9 [99.8, >99.9] | >99.9 [>99.9, >99.9] | >99.9 [>99.9, >99.9] | >99.9 [>99.9, >99.9] | >99.9 [>99.9, >99.9] |
| 63 | >99.9 [>99.9, >99.9] | >99.9 [>99.9, >99.9] | >99.9 [99.9, >99.9] | >99.9 [>99.9, >99.9] | >99.9 [>99.9, >99.9] | >99.9 [>99.9, >99.9] | >99.9 [>99.9, >99.9] |
| 64 | >99.9 [>99.9, >99.9] | >99.9 [>99.9, >99.9] | >99.9 [99.9, >99.9] | >99.9 [>99.9, >99.9] | >99.9 [>99.9, >99.9] | >99.9 [>99.9, >99.9] | >99.9 [>99.9, >99.9] |
| 65 | >99.9 [>99.9, >99.9] | >99.9 [>99.9, >99.9] | >99.9 [>99.9, >99.9] | >99.9 [>99.9, >99.9] | >99.9 [>99.9, >99.9] | >99.9 [>99.9, >99.9] | >99.9 [>99.9, >99.9] |
| 66 | >99.9 [>99.9, >99.9] | >99.9 [>99.9, >99.9] | >99.9 [>99.9, >99.9] | >99.9 [>99.9, >99.9] | >99.9 [>99.9, >99.9] | >99.9 [>99.9, >99.9] | >99.9 [>99.9, >99.9] |
| 67 | >99.9 [>99.9, >99.9] | >99.9 [>99.9, >99.9] | >99.9 [>99.9, >99.9] | >99.9 [>99.9, >99.9] | >99.9 [>99.9, >99.9] | >99.9 [>99.9, >99.9] | >99.9 [>99.9, >99.9] |
| 68 | >99.9 [>99.9, >99.9] | >99.9 [>99.9, >99.9] | >99.9 [>99.9, >99.9] | >99.9 [>99.9, >99.9] | >99.9 [>99.9, >99.9] | >99.9 [>99.9, >99.9] | >99.9 [>99.9, >99.9] |
| 69 | >99.9 [>99.9, >99.9] | >99.9 [>99.9, >99.9] | >99.9 [>99.9, >99.9] | >99.9 [>99.9, >99.9] | >99.9 [>99.9, >99.9] | >99.9 [>99.9, >99.9] | >99.9 [>99.9, >99.9] |
| 70 | >99.9 [>99.9, >99.9] | >99.9 [>99.9, >99.9] | >99.9 [>99.9, >99.9] | >99.9 [>99.9, >99.9] | >99.9 [>99.9, >99.9] | >99.9 [>99.9, >99.9] | >99.9 [>99.9, >99.9] |
| 71 | >99.9 [>99.9, >99.9] | >99.9 [>99.9, >99.9] | >99.9 [>99.9, >99.9] | >99.9 [>99.9, >99.9] | >99.9 [>99.9, >99.9] | >99.9 [>99.9, >99.9] | >99.9 [>99.9, >99.9] |
| 72 | >99.9 [>99.9, >99.9] | >99.9 [>99.9, >99.9] | >99.9 [>99.9, >99.9] | >99.9 [>99.9, >99.9] | >99.9 [>99.9, >99.9] | >99.9 [>99.9, >99.9] | >99.9 [>99.9, >99.9] |

*Note. Smoothed cumulative percentiles (SCAM). 95% CI = bootstrap (B = 1,000).*

*Supplementary Table S10. SOM norms - Men by age group.*

| Score | 16-24 [95% CI] | 25-34 [95% CI] | 35-44 [95% CI] | 45-54 [95% CI] | 55-64 [95% CI] | 65-74 [95% CI] | 75+ [95% CI] |
| --- | --- | --- | --- | --- | --- | --- | --- |
| 0 | 80.6 [72.6, 88.3] | 81.9 [76.3, 87.3] | 80.8 [75.2, 86.0] | 68.3 [61.6, 74.7] | 62.8 [57.0, 68.5] | 56.5 [49.5, 63.8] | 46.3 [35.9, 56.4] |
| 1 | 88.5 [82.4, 93.6] | 89.4 [85.1, 93.0] | 89.1 [84.8, 92.8] | 79.1 [73.4, 84.3] | 76.2 [71.1, 80.7] | 71.5 [65.2, 77.6] | 61.8 [52.9, 70.4] |
| 2 | 93.5 [88.6, 97.5] | 93.2 [89.5, 96.4] | 93.1 [89.0, 96.6] | 86.2 [81.2, 90.7] | 83.9 [79.1, 88.0] | 80.9 [75.1, 86.2] | 72.5 [64.5, 80.4] |
| 3 | 96.5 [93.4, 99.0] | 94.9 [91.6, 97.7] | 94.7 [91.3, 97.9] | 90.6 [86.6, 94.2] | 88.0 [84.0, 91.7] | 86.4 [81.4, 90.7] | 79.8 [72.1, 86.6] |
| 4 | 98.3 [96.3, 99.5] | 96.0 [93.0, 98.4] | 95.7 [92.8, 98.4] | 93.5 [89.9, 96.6] | 90.5 [86.9, 93.7] | 89.9 [85.4, 93.6] | 85.2 [78.1, 91.3] |
| 5 | 99.3 [98.0, 99.9] | 96.9 [94.3, 99.0] | 96.9 [94.4, 99.0] | 95.3 [92.0, 97.9] | 92.4 [89.2, 95.3] | 92.3 [88.4, 95.6] | 89.5 [83.4, 94.8] |
| 6 | 99.7 [99.0, >99.9] | 97.7 [95.7, 99.4] | 98.0 [96.1, 99.4] | 96.3 [93.6, 98.5] | 93.8 [90.8, 96.5] | 93.9 [90.5, 96.9] | 92.8 [88.0, 96.8] |
| 7 | 99.9 [99.6, >99.9] | 98.4 [96.6, 99.7] | 98.9 [97.4, 99.7] | 96.9 [94.2, 98.9] | 95.0 [92.2, 97.4] | 95.0 [91.7, 97.8] | 95.1 [91.3, 98.3] |
| 8 | >99.9 [99.9, >99.9] | 98.9 [97.4, 99.9] | 99.5 [98.4, 99.9] | 97.2 [94.7, 99.1] | 95.9 [93.4, 98.1] | 95.9 [92.9, 98.5] | 96.7 [93.4, 99.3] |
| 9 | >99.9 [>99.9, >99.9] | 99.2 [97.9, >99.9] | 99.8 [99.1, >99.9] | 97.4 [94.9, 99.4] | 96.7 [94.3, 98.6] | 96.7 [94.1, 98.9] | 97.7 [94.7, 99.9] |
| 10 | >99.9 [>99.9, >99.9] | 99.5 [98.3, >99.9] | 99.9 [99.6, >99.9] | 97.6 [95.3, 99.5] | 97.2 [95.1, 98.8] | 97.4 [95.2, 99.3] | 98.3 [95.6, >99.9] |
| 11 | >99.9 [>99.9, >99.9] | 99.6 [98.7, >99.9] | 99.9 [99.9, >99.9] | 97.9 [95.9, 99.6] | 97.6 [95.6, 99.1] | 98.0 [96.3, 99.5] | 98.6 [95.9, >99.9] |
| 12 | >99.9 [>99.9, >99.9] | 99.7 [99.1, >99.9] | >99.9 [99.9, >99.9] | 98.3 [96.5, 99.7] | 97.8 [95.9, 99.3] | 98.7 [97.5, 99.8] | 98.9 [96.1, >99.9] |
| 13 | >99.9 [>99.9, >99.9] | 99.8 [99.4, >99.9] | >99.9 [>99.9, >99.9] | 98.7 [97.1, 99.8] | 97.9 [96.2, 99.5] | 99.3 [98.6, 99.9] | 99.1 [96.3, >99.9] |
| 14 | >99.9 [>99.9, >99.9] | 99.9 [99.7, >99.9] | >99.9 [>99.9, >99.9] | 99.1 [97.7, 99.9] | 98.2 [96.6, 99.6] | 99.7 [99.3, >99.9] | 99.2 [96.7, >99.9] |
| 15 | >99.9 [>99.9, >99.9] | 99.9 [99.8, >99.9] | >99.9 [>99.9, >99.9] | 99.5 [98.4, >99.9] | 98.5 [97.1, 99.7] | >99.9 [99.6, >99.9] | 99.3 [96.9, >99.9] |
| 16 | >99.9 [>99.9, >99.9] | 99.9 [99.8, >99.9] | >99.9 [>99.9, >99.9] | 99.7 [99.1, >99.9] | 98.9 [97.7, 99.7] | >99.9 [99.8, >99.9] | 99.4 [97.1, >99.9] |
| 17 | >99.9 [>99.9, >99.9] | >99.9 [99.9, >99.9] | >99.9 [>99.9, >99.9] | 99.9 [99.6, >99.9] | 99.2 [98.0, 99.9] | >99.9 [99.9, >99.9] | 99.5 [97.4, >99.9] |
| 18 | >99.9 [>99.9, >99.9] | >99.9 [>99.9, >99.9] | >99.9 [>99.9, >99.9] | >99.9 [99.8, >99.9] | 99.4 [98.3, >99.9] | >99.9 [>99.9, >99.9] | 99.6 [98.0, >99.9] |
| 19 | >99.9 [>99.9, >99.9] | >99.9 [>99.9, >99.9] | >99.9 [>99.9, >99.9] | >99.9 [99.9, >99.9] | 99.5 [98.5, >99.9] | >99.9 [>99.9, >99.9] | 99.7 [98.6, >99.9] |
| 20 | >99.9 [>99.9, >99.9] | >99.9 [>99.9, >99.9] | >99.9 [>99.9, >99.9] | >99.9 [>99.9, >99.9] | 99.6 [98.7, >99.9] | >99.9 [>99.9, >99.9] | 99.8 [99.2, >99.9] |
| 21 | >99.9 [>99.9, >99.9] | >99.9 [>99.9, >99.9] | >99.9 [>99.9, >99.9] | >99.9 [>99.9, >99.9] | 99.6 [98.8, >99.9] | >99.9 [>99.9, >99.9] | 99.9 [99.6, >99.9] |
| 22 | >99.9 [>99.9, >99.9] | >99.9 [>99.9, >99.9] | >99.9 [>99.9, >99.9] | >99.9 [>99.9, >99.9] | 99.6 [98.9, >99.9] | >99.9 [>99.9, >99.9] | >99.9 [99.8, >99.9] |
| 23 | >99.9 [>99.9, >99.9] | >99.9 [>99.9, >99.9] | >99.9 [>99.9, >99.9] | >99.9 [>99.9, >99.9] | 99.7 [99.2, >99.9] | >99.9 [>99.9, >99.9] | >99.9 [>99.9, >99.9] |
| 24 | >99.9 [>99.9, >99.9] | >99.9 [>99.9, >99.9] | >99.9 [>99.9, >99.9] | >99.9 [>99.9, >99.9] | 99.9 [99.4, >99.9] | >99.9 [>99.9, >99.9] | >99.9 [>99.9, >99.9] |

*Note. Smoothed cumulative percentiles (SCAM). 95% CI = bootstrap (B = 1,000).*

*Supplementary Table S11. SOM norms – Women by age group.*

| Score | 16-24 [95% CI] | 25-34 [95% CI] | 35-44 [95% CI] | 45-54 [95% CI] | 55-64 [95% CI] | 65-74 [95% CI] | 75+ [95% CI] |
| --- | --- | --- | --- | --- | --- | --- | --- |
| 0 | 69.6 [61.5, 78.3] | 68.9 [62.5, 75.8] | 67.8 [61.9, 73.8] | 71.9 [66.1, 77.6] | 62.2 [56.0, 68.0] | 54.4 [47.6, 60.8] | 36.9 [28.6, 45.7] |
| 1 | 81.0 [74.5, 87.4] | 78.8 [72.7, 84.6] | 79.7 [74.6, 84.6] | 82.9 [78.1, 87.2] | 74.0 [68.7, 79.1] | 66.9 [60.2, 72.8] | 53.4 [45.5, 61.3] |
| 2 | 88.3 [82.5, 93.3] | 85.5 [80.1, 90.3] | 86.1 [81.5, 90.5] | 88.6 [84.3, 92.5] | 81.5 [76.4, 85.9] | 76.7 [70.9, 82.0] | 64.7 [57.2, 72.6] |
| 3 | 92.6 [88.3, 96.3] | 89.8 [85.3, 93.9] | 89.2 [85.3, 92.9] | 91.1 [87.2, 94.6] | 86.0 [81.4, 89.9] | 84.2 [79.3, 88.5] | 72.6 [65.1, 79.9] |
| 4 | 95.3 [91.8, 98.0] | 92.6 [88.8, 96.0] | 91.1 [87.4, 94.3] | 92.6 [88.8, 95.7] | 89.0 [85.0, 92.6] | 89.5 [85.1, 93.3] | 78.7 [71.5, 85.0] |
| 5 | 96.9 [93.7, 99.4] | 94.6 [91.3, 97.5] | 92.6 [89.4, 95.6] | 93.9 [90.5, 96.8] | 91.1 [87.6, 94.5] | 93.1 [89.4, 96.2] | 83.8 [77.7, 89.1] |
| 6 | 97.9 [95.2, 99.9] | 96.0 [93.0, 98.4] | 93.9 [90.9, 96.6] | 95.0 [92.1, 97.6] | 92.8 [89.5, 95.9] | 95.4 [92.4, 97.7] | 88.0 [83.0, 92.3] |
| 7 | 98.5 [96.1, >99.9] | 97.0 [94.5, 99.1] | 95.0 [92.4, 97.5] | 96.1 [93.6, 98.2] | 94.2 [91.1, 96.9] | 96.8 [94.4, 98.6] | 91.4 [86.9, 95.1] |
| 8 | 99.0 [96.9, >99.9] | 97.9 [95.7, 99.5] | 96.0 [93.5, 98.2] | 97.1 [95.0, 98.8] | 95.4 [92.8, 97.8] | 97.8 [95.7, 99.3] | 94.0 [90.2, 97.3] |
| 9 | 99.3 [97.8, >99.9] | 98.6 [96.9, 99.8] | 96.9 [94.8, 98.7] | 97.9 [96.1, 99.3] | 96.5 [94.2, 98.4] | 98.4 [96.6, 99.7] | 96.1 [93.0, 98.5] |
| 10 | 99.5 [98.5, >99.9] | 99.2 [97.9, 99.9] | 97.6 [95.8, 99.2] | 98.6 [97.0, 99.7] | 97.4 [95.4, 99.0] | 98.8 [97.4, 99.9] | 97.5 [95.2, 99.2] |
| 11 | 99.7 [99.0, >99.9] | 99.6 [98.6, >99.9] | 98.2 [96.6, 99.5] | 99.0 [97.6, 99.9] | 98.2 [96.5, 99.5] | 99.1 [97.9, >99.9] | 98.5 [96.5, 99.7] |
| 12 | 99.8 [99.3, >99.9] | 99.8 [99.1, >99.9] | 98.6 [97.3, 99.8] | 99.3 [98.0, >99.9] | 98.8 [97.5, 99.8] | 99.3 [98.2, >99.9] | 99.0 [97.5, >99.9] |
| 13 | 99.9 [99.6, >99.9] | 99.9 [99.5, >99.9] | 99.0 [97.7, 99.9] | 99.5 [98.3, >99.9] | 99.2 [98.3, 99.9] | 99.5 [98.5, >99.9] | 99.4 [98.2, >99.9] |
| 14 | 99.9 [99.8, >99.9] | 99.9 [99.7, >99.9] | 99.2 [98.1, >99.9] | 99.6 [98.7, >99.9] | 99.5 [98.8, >99.9] | 99.6 [98.8, >99.9] | 99.6 [98.6, >99.9] |
| 15 | >99.9 [99.8, >99.9] | >99.9 [99.8, >99.9] | 99.4 [98.5, >99.9] | 99.7 [99.1, >99.9] | 99.7 [99.1, >99.9] | 99.7 [99.1, >99.9] | 99.7 [99.0, >99.9] |
| 16 | >99.9 [99.9, >99.9] | >99.9 [99.9, >99.9] | 99.6 [98.9, >99.9] | 99.8 [99.5, >99.9] | 99.8 [99.4, >99.9] | 99.8 [99.3, >99.9] | 99.8 [99.3, >99.9] |
| 17 | >99.9 [>99.9, >99.9] | >99.9 [>99.9, >99.9] | 99.7 [99.3, >99.9] | 99.9 [99.7, >99.9] | 99.9 [99.6, >99.9] | 99.9 [99.5, >99.9] | 99.9 [99.6, >99.9] |
| 18 | >99.9 [>99.9, >99.9] | >99.9 [>99.9, >99.9] | 99.8 [99.5, >99.9] | 99.9 [99.8, >99.9] | 99.9 [99.8, >99.9] | 99.9 [99.7, >99.9] | >99.9 [99.8, >99.9] |
| 19 | >99.9 [>99.9, >99.9] | >99.9 [>99.9, >99.9] | 99.9 [99.7, >99.9] | >99.9 [99.9, >99.9] | >99.9 [99.9, >99.9] | >99.9 [99.8, >99.9] | >99.9 [99.9, >99.9] |
| 20 | >99.9 [>99.9, >99.9] | >99.9 [>99.9, >99.9] | >99.9 [99.9, >99.9] | >99.9 [>99.9, >99.9] | >99.9 [>99.9, >99.9] | >99.9 [99.9, >99.9] | >99.9 [>99.9, >99.9] |
| 21 | >99.9 [>99.9, >99.9] | >99.9 [>99.9, >99.9] | >99.9 [>99.9, >99.9] | >99.9 [>99.9, >99.9] | >99.9 [>99.9, >99.9] | >99.9 [>99.9, >99.9] | >99.9 [>99.9, >99.9] |
| 22 | >99.9 [>99.9, >99.9] | >99.9 [>99.9, >99.9] | >99.9 [>99.9, >99.9] | >99.9 [>99.9, >99.9] | >99.9 [>99.9, >99.9] | >99.9 [>99.9, >99.9] | >99.9 [>99.9, >99.9] |
| 23 | >99.9 [>99.9, >99.9] | >99.9 [>99.9, >99.9] | >99.9 [>99.9, >99.9] | >99.9 [>99.9, >99.9] | >99.9 [>99.9, >99.9] | >99.9 [>99.9, >99.9] | >99.9 [>99.9, >99.9] |
| 24 | >99.9 [>99.9, >99.9] | >99.9 [>99.9, >99.9] | >99.9 [>99.9, >99.9] | >99.9 [>99.9, >99.9] | >99.9 [>99.9, >99.9] | >99.9 [>99.9, >99.9] | >99.9 [>99.9, >99.9] |

*Note. Smoothed cumulative percentiles (SCAM). 95% CI = bootstrap (B = 1,000).*

*Supplementary Table S12. DEP norms - Men by age group.*

| Score | 16-24 [95% CI] | 25-34 [95% CI] | 35-44 [95% CI] | 45-54 [95% CI] | 55-64 [95% CI] | 65-74 [95% CI] | 75+ [95% CI] |
| --- | --- | --- | --- | --- | --- | --- | --- |
| 0 | 59.4 [49.7, 68.5] | 72.0 [65.5, 78.2] | 65.5 [58.3, 72.4] | 60.8 [54.2, 67.6] | 55.5 [49.1, 61.5] | 46.2 [38.2, 53.2] | 42.4 [33.5, 52.0] |
| 1 | 70.8 [61.6, 78.5] | 83.5 [78.8, 88.1] | 79.3 [73.8, 84.4] | 71.3 [65.1, 77.5] | 67.5 [61.8, 72.7] | 64.4 [58.0, 70.3] | 58.2 [49.5, 67.2] |
| 2 | 77.5 [68.8, 84.7] | 89.8 [85.5, 93.8] | 87.3 [82.5, 91.6] | 78.2 [72.2, 83.8] | 76.3 [71.1, 81.3] | 75.5 [69.4, 81.0] | 70.0 [61.2, 78.4] |
| 3 | 81.7 [74.2, 88.5] | 92.9 [89.3, 96.1] | 91.4 [87.2, 94.8] | 82.7 [77.7, 87.9] | 82.4 [77.7, 86.4] | 81.6 [75.9, 86.2] | 78.2 [70.4, 85.6] |
| 4 | 85.5 [79.2, 91.5] | 94.7 [91.4, 97.3] | 93.6 [90.0, 96.7] | 86.4 [81.8, 91.0] | 86.3 [82.0, 90.0] | 85.1 [79.7, 89.7] | 83.2 [75.6, 89.6] |
| 5 | 89.8 [84.7, 94.6] | 96.0 [93.1, 98.3] | 95.0 [91.6, 97.9] | 89.7 [85.5, 93.3] | 88.5 [84.2, 92.1] | 87.5 [82.5, 91.7] | 85.9 [78.9, 92.2] |
| 6 | 93.8 [90.0, 96.8] | 96.9 [94.5, 98.9] | 95.8 [92.6, 98.4] | 92.5 [89.1, 95.6] | 89.9 [85.9, 93.4] | 89.2 [84.6, 93.2] | 87.2 [80.7, 93.4] |
| 7 | 96.9 [94.1, 98.5] | 97.6 [95.6, 99.3] | 96.3 [93.3, 98.7] | 94.7 [91.8, 97.3] | 90.9 [87.1, 94.2] | 90.8 [86.8, 94.5] | 88.0 [82.2, 94.2] |
| 8 | 98.7 [96.3, 99.6] | 98.1 [96.3, 99.7] | 96.7 [93.9, 99.0] | 96.2 [93.7, 98.5] | 92.0 [88.6, 95.2] | 92.8 [89.3, 95.9] | 89.1 [83.8, 95.0] |
| 9 | 99.5 [97.6, >99.9] | 98.5 [96.9, 99.9] | 97.1 [94.6, 99.2] | 97.2 [95.0, 99.1] | 93.2 [90.1, 96.1] | 94.8 [91.8, 97.2] | 90.5 [85.3, 95.8] |
| 10 | 99.7 [98.4, >99.9] | 98.8 [97.2, 99.9] | 97.5 [95.2, 99.4] | 97.9 [95.7, 99.5] | 94.3 [91.3, 96.8] | 96.5 [94.0, 98.2] | 92.2 [87.4, 96.8] |
| 11 | 99.8 [98.8, >99.9] | 99.0 [97.5, >99.9] | 97.9 [95.8, 99.5] | 98.3 [96.3, 99.8] | 95.2 [92.3, 97.5] | 97.6 [95.2, 99.1] | 94.1 [89.9, 97.9] |
| 12 | 99.9 [99.2, >99.9] | 99.1 [97.6, >99.9] | 98.3 [96.2, 99.7] | 98.7 [96.8, >99.9] | 96.0 [93.1, 98.0] | 98.2 [95.8, 99.6] | 96.1 [93.0, 98.7] |
| 13 | 99.9 [99.5, >99.9] | 99.3 [97.8, >99.9] | 98.7 [96.8, 99.9] | 98.9 [97.3, >99.9] | 96.5 [93.9, 98.4] | 98.4 [96.2, 99.8] | 97.8 [96.0, 99.3] |
| 14 | 99.9 [99.7, >99.9] | 99.4 [98.0, >99.9] | 99.0 [97.3, 99.9] | 99.1 [97.7, >99.9] | 96.9 [94.6, 98.7] | 98.5 [96.6, 99.9] | 99.1 [98.1, 99.7] |
| 15 | >99.9 [99.8, >99.9] | 99.5 [98.3, >99.9] | 99.3 [98.0, >99.9] | 99.3 [98.2, >99.9] | 97.2 [95.1, 99.0] | 98.8 [97.1, >99.9] | 99.8 [99.2, >99.9] |
| 16 | >99.9 [99.9, >99.9] | 99.6 [98.7, >99.9] | 99.5 [98.7, >99.9] | 99.5 [98.6, >99.9] | 97.5 [95.6, 99.2] | 99.0 [97.7, >99.9] | >99.9 [99.6, >99.9] |
| 17 | >99.9 [>99.9, >99.9] | 99.7 [99.0, >99.9] | 99.7 [99.2, >99.9] | 99.6 [99.0, >99.9] | 97.8 [96.0, 99.3] | 99.3 [98.3, >99.9] | >99.9 [99.8, >99.9] |
| 18 | >99.9 [>99.9, >99.9] | 99.8 [99.3, >99.9] | 99.8 [99.6, >99.9] | 99.7 [99.3, >99.9] | 98.1 [96.3, 99.5] | 99.6 [98.9, >99.9] | >99.9 [>99.9, >99.9] |
| 19 | >99.9 [>99.9, >99.9] | 99.8 [99.5, >99.9] | 99.9 [99.7, >99.9] | 99.8 [99.5, >99.9] | 98.4 [96.7, 99.6] | 99.8 [99.4, >99.9] | >99.9 [>99.9, >99.9] |
| 20 | >99.9 [>99.9, >99.9] | 99.9 [99.8, >99.9] | >99.9 [99.9, >99.9] | 99.9 [99.7, >99.9] | 98.7 [97.2, 99.8] | 99.9 [99.7, >99.9] | >99.9 [>99.9, >99.9] |
| 21 | >99.9 [>99.9, >99.9] | >99.9 [99.9, >99.9] | >99.9 [>99.9, >99.9] | >99.9 [99.9, >99.9] | 99.0 [97.8, 99.9] | >99.9 [99.9, >99.9] | >99.9 [>99.9, >99.9] |
| 22 | >99.9 [>99.9, >99.9] | >99.9 [>99.9, >99.9] | >99.9 [>99.9, >99.9] | >99.9 [>99.9, >99.9] | 99.4 [98.4, >99.9] | >99.9 [>99.9, >99.9] | >99.9 [>99.9, >99.9] |
| 23 | >99.9 [>99.9, >99.9] | >99.9 [>99.9, >99.9] | >99.9 [>99.9, >99.9] | >99.9 [>99.9, >99.9] | 99.7 [99.1, >99.9] | >99.9 [>99.9, >99.9] | >99.9 [>99.9, >99.9] |
| 24 | >99.9 [>99.9, >99.9] | >99.9 [>99.9, >99.9] | >99.9 [>99.9, >99.9] | >99.9 [>99.9, >99.9] | >99.9 [99.6, >99.9] | >99.9 [>99.9, >99.9] | >99.9 [>99.9, >99.9] |

*Note. Smoothed cumulative percentiles (SCAM). 95% CI = bootstrap (B = 1,000).*

*Supplementary Table S13. DEP norms - Women by age group.*

| Score | 16-24 [95% CI] | 25-34 [95% CI] | 35-44 [95% CI] | 45-54 [95% CI] | 55-64 [95% CI] | 65-74 [95% CI] | 75+ [95% CI] |
| --- | --- | --- | --- | --- | --- | --- | --- |
| 0 | 47.6 [39.3, 56.9] | 62.1 [54.4, 69.2] | 59.0 [52.5, 65.2] | 54.7 [48.2, 61.3] | 48.7 [42.4, 54.8] | 47.0 [39.8, 53.7] | 32.1 [24.7, 39.8] |
| 1 | 66.1 [58.0, 73.3] | 68.4 [62.0, 75.1] | 70.2 [64.1, 75.7] | 69.6 [63.5, 75.2] | 62.5 [57.3, 68.3] | 61.3 [54.7, 67.4] | 49.1 [41.0, 57.0] |
| 2 | 75.4 [67.7, 82.7] | 75.9 [70.2, 81.3] | 77.9 [72.4, 82.6] | 77.8 [71.9, 83.2] | 71.5 [66.4, 77.0] | 71.2 [65.0, 76.6] | 61.4 [53.4, 69.5] |
| 3 | 79.6 [72.8, 86.5] | 82.9 [77.6, 87.2] | 82.9 [78.1, 87.1] | 82.0 [76.6, 86.9] | 77.5 [72.6, 82.2] | 77.9 [72.4, 82.7] | 70.3 [63.3, 77.1] |
| 4 | 82.9 [76.8, 89.3] | 88.0 [82.9, 91.9] | 86.1 [81.8, 90.3] | 84.8 [80.0, 89.4] | 82.0 [77.2, 86.1] | 82.9 [77.8, 87.4] | 76.8 [70.2, 82.7] |
| 5 | 86.7 [81.4, 91.9] | 90.8 [86.1, 94.5] | 88.4 [84.4, 92.4] | 87.5 [83.3, 91.6] | 85.7 [81.6, 89.5] | 87.1 [82.9, 91.2] | 81.7 [75.5, 87.2] |
| 6 | 90.4 [85.6, 94.4] | 92.1 [88.0, 95.9] | 89.9 [86.2, 93.6] | 90.0 [86.1, 93.7] | 88.8 [85.1, 92.2] | 90.4 [86.7, 93.8] | 85.1 [79.3, 90.2] |
| 7 | 93.3 [88.7, 96.5] | 93.1 [89.1, 96.6] | 91.2 [87.7, 94.7] | 92.3 [88.6, 95.6] | 91.3 [88.0, 94.5] | 93.0 [89.4, 95.9] | 87.3 [81.8, 92.3] |
| 8 | 94.9 [90.6, 98.0] | 94.1 [90.5, 97.2] | 92.6 [89.4, 95.8] | 94.3 [91.3, 97.0] | 93.3 [90.1, 96.2] | 94.9 [91.7, 97.4] | 88.5 [82.9, 93.6] |
| 9 | 95.7 [91.7, 98.7] | 95.3 [92.1, 97.8] | 93.9 [91.1, 96.8] | 96.0 [93.3, 98.2] | 94.8 [92.0, 97.2] | 96.2 [93.5, 98.3] | 89.3 [83.8, 94.4] |
| 10 | 96.1 [92.5, 99.1] | 96.3 [93.3, 98.5] | 95.1 [92.4, 97.5] | 97.4 [95.1, 99.0] | 96.0 [93.4, 98.0] | 97.2 [94.9, 98.9] | 90.2 [85.0, 94.9] |
| 11 | 96.5 [93.4, 99.4] | 97.0 [94.2, 99.1] | 96.0 [93.5, 98.1] | 98.3 [96.3, 99.5] | 96.9 [94.5, 98.6] | 97.9 [95.9, 99.4] | 91.5 [87.0, 95.7] |
| 12 | 97.2 [94.4, 99.5] | 97.4 [94.9, 99.5] | 96.7 [94.5, 98.6] | 98.9 [97.2, 99.9] | 97.6 [95.6, 99.1] | 98.5 [96.7, 99.7] | 93.3 [89.6, 96.8] |
| 13 | 97.9 [95.3, 99.7] | 97.7 [95.5, 99.6] | 97.3 [95.1, 99.0] | 99.2 [97.8, >99.9] | 98.2 [96.5, 99.5] | 98.8 [97.3, 99.9] | 95.3 [92.0, 98.0] |
| 14 | 98.5 [96.3, 99.8] | 98.1 [96.2, 99.8] | 97.7 [95.7, 99.3] | 99.4 [98.3, >99.9] | 98.6 [97.2, 99.8] | 99.1 [97.7, 99.9] | 96.9 [94.1, 99.0] |
| 15 | 99.1 [97.5, 99.9] | 98.6 [97.2, 99.9] | 98.1 [96.2, 99.5] | 99.6 [98.7, >99.9] | 98.9 [97.7, 99.9] | 99.3 [98.2, >99.9] | 98.2 [96.1, 99.7] |
| 16 | 99.5 [98.5, >99.9] | 99.1 [98.1, >99.9] | 98.4 [96.6, 99.7] | 99.7 [99.2, >99.9] | 99.2 [98.1, >99.9] | 99.5 [98.7, >99.9] | 99.1 [97.7, >99.9] |
| 17 | 99.8 [99.3, >99.9] | 99.6 [98.7, >99.9] | 98.7 [97.1, 99.8] | 99.8 [99.5, >99.9] | 99.4 [98.4, >99.9] | 99.6 [99.0, >99.9] | 99.6 [99.0, >99.9] |
| 18 | >99.9 [99.7, >99.9] | 99.9 [99.2, >99.9] | 98.9 [97.6, 99.9] | 99.9 [99.7, >99.9] | 99.5 [98.8, >99.9] | 99.8 [99.3, >99.9] | 99.9 [99.6, >99.9] |
| 19 | >99.9 [99.9, >99.9] | >99.9 [99.5, >99.9] | 99.2 [98.0, >99.9] | 99.9 [99.8, >99.9] | 99.7 [99.2, >99.9] | 99.9 [99.5, >99.9] | >99.9 [99.8, >99.9] |
| 20 | >99.9 [>99.9, >99.9] | >99.9 [99.8, >99.9] | 99.4 [98.3, >99.9] | >99.9 [99.9, >99.9] | 99.8 [99.5, >99.9] | >99.9 [99.7, >99.9] | >99.9 [>99.9, >99.9] |
| 21 | >99.9 [>99.9, >99.9] | >99.9 [>99.9, >99.9] | 99.5 [98.6, >99.9] | >99.9 [>99.9, >99.9] | 99.9 [99.7, >99.9] | >99.9 [99.9, >99.9] | >99.9 [>99.9, >99.9] |
| 22 | >99.9 [>99.9, >99.9] | >99.9 [>99.9, >99.9] | 99.7 [98.7, >99.9] | >99.9 [>99.9, >99.9] | >99.9 [99.9, >99.9] | >99.9 [>99.9, >99.9] | >99.9 [>99.9, >99.9] |
| 23 | >99.9 [>99.9, >99.9] | >99.9 [>99.9, >99.9] | 99.8 [99.0, >99.9] | >99.9 [>99.9, >99.9] | >99.9 [>99.9, >99.9] | >99.9 [>99.9, >99.9] | >99.9 [>99.9, >99.9] |
| 24 | >99.9 [>99.9, >99.9] | >99.9 [>99.9, >99.9] | >99.9 [99.4, >99.9] | >99.9 [>99.9, >99.9] | >99.9 [>99.9, >99.9] | >99.9 [>99.9, >99.9] | >99.9 [>99.9, >99.9] |

*Note. Smoothed cumulative percentiles (SCAM). 95% CI = bootstrap (B = 1,000).*

*Supplementary Table S14. ANX norms - Men by age group.*

| Score | 16-24 [95% CI] | 25-34 [95% CI] | 35-44 [95% CI] | 45-54 [95% CI] | 55-64 [95% CI] | 65-74 [95% CI] | 75+ [95% CI] |
| --- | --- | --- | --- | --- | --- | --- | --- |
| 0 | 66.6 [57.6, 75.3] | 74.0 [67.9, 79.6] | 67.8 [60.9, 75.0] | 60.1 [53.0, 66.8] | 62.4 [56.2, 68.4] | 60.4 [53.5, 67.2] | 66.1 [56.6, 75.5] |
| 1 | 83.5 [77.3, 88.9] | 85.9 [81.0, 89.5] | 85.1 [80.7, 89.0] | 73.2 [67.2, 78.7] | 75.6 [70.2, 80.8] | 76.7 [71.2, 81.7] | 76.9 [69.2, 84.6] |
| 2 | 92.2 [87.2, 96.8] | 91.8 [87.5, 95.2] | 92.8 [88.7, 96.2] | 82.7 [77.5, 87.1] | 83.9 [79.2, 88.4] | 85.5 [80.2, 89.9] | 85.0 [78.0, 91.0] |
| 3 | 96.0 [92.3, 99.1] | 94.3 [90.9, 97.1] | 95.0 [91.4, 98.1] | 89.2 [85.2, 92.4] | 88.7 [84.9, 92.4] | 89.5 [85.0, 93.5] | 90.5 [85.4, 94.8] |
| 4 | 97.5 [93.9, 99.5] | 95.5 [92.7, 98.0] | 95.7 [92.5, 98.4] | 93.1 [89.6, 95.7] | 91.3 [87.8, 94.7] | 91.5 [87.5, 95.2] | 93.9 [89.8, 97.4] |
| 5 | 98.2 [95.1, 99.9] | 96.7 [94.3, 98.7] | 96.5 [93.9, 98.8] | 95.1 [91.9, 97.5] | 92.7 [89.3, 96.0] | 92.9 [89.1, 96.2] | 95.7 [91.7, 99.1] |
| 6 | 98.6 [96.1, >99.9] | 97.8 [95.7, 99.3] | 97.4 [95.1, 99.2] | 95.9 [93.0, 98.3] | 93.3 [90.1, 96.7] | 93.9 [90.4, 97.0] | 96.5 [92.6, 99.8] |
| 7 | 98.8 [97.0, >99.9] | 98.5 [96.6, 99.7] | 98.3 [96.1, 99.6] | 96.2 [93.5, 98.6] | 93.9 [91.2, 97.1] | 94.8 [91.4, 97.5] | 97.0 [93.5, 99.9] |
| 8 | 99.1 [97.8, >99.9] | 98.9 [97.1, 99.9] | 98.9 [97.1, 99.9] | 96.6 [94.4, 98.8] | 94.8 [92.4, 97.6] | 95.7 [92.5, 98.1] | 97.7 [95.1, 99.9] |
| 9 | 99.5 [98.6, >99.9] | 99.1 [97.4, >99.9] | 99.3 [98.1, >99.9] | 97.2 [95.1, 99.1] | 95.8 [93.6, 98.1] | 96.6 [93.7, 98.6] | 98.5 [96.7, >99.9] |
| 10 | 99.7 [99.3, >99.9] | 99.2 [97.7, >99.9] | 99.7 [98.9, >99.9] | 97.8 [95.8, 99.4] | 96.8 [94.8, 98.7] | 97.4 [94.8, 99.1] | 99.2 [98.2, >99.9] |
| 11 | 99.9 [99.8, >99.9] | 99.4 [98.2, >99.9] | 99.8 [99.3, >99.9] | 98.4 [96.7, 99.7] | 97.7 [95.6, 99.2] | 98.1 [96.0, 99.5] | 99.7 [99.0, >99.9] |
| 12 | >99.9 [99.8, >99.9] | 99.6 [98.8, >99.9] | 99.9 [99.6, >99.9] | 99.0 [97.7, 99.9] | 98.3 [96.4, 99.5] | 98.7 [97.1, 99.8] | 99.9 [99.5, >99.9] |
| 13 | >99.9 [99.9, >99.9] | 99.8 [99.3, >99.9] | >99.9 [99.8, >99.9] | 99.4 [98.7, >99.9] | 98.7 [97.1, 99.7] | 99.1 [98.1, 99.9] | >99.9 [99.8, >99.9] |
| 14 | >99.9 [99.9, >99.9] | 99.9 [99.6, >99.9] | >99.9 [99.9, >99.9] | 99.8 [99.3, >99.9] | 99.0 [97.5, 99.9] | 99.5 [98.8, >99.9] | >99.9 [99.9, >99.9] |
| 15 | >99.9 [>99.9, >99.9] | >99.9 [99.8, >99.9] | >99.9 [>99.9, >99.9] | 99.9 [99.6, >99.9] | 99.1 [97.9, >99.9] | 99.7 [99.2, >99.9] | >99.9 [99.9, >99.9] |
| 16 | >99.9 [>99.9, >99.9] | >99.9 [99.8, >99.9] | >99.9 [>99.9, >99.9] | >99.9 [99.8, >99.9] | 99.2 [98.0, >99.9] | 99.8 [99.5, >99.9] | >99.9 [>99.9, >99.9] |
| 17 | >99.9 [>99.9, >99.9] | >99.9 [99.9, >99.9] | >99.9 [>99.9, >99.9] | >99.9 [99.9, >99.9] | 99.2 [98.2, >99.9] | 99.9 [99.7, >99.9] | >99.9 [>99.9, >99.9] |
| 18 | >99.9 [>99.9, >99.9] | >99.9 [>99.9, >99.9] | >99.9 [>99.9, >99.9] | >99.9 [>99.9, >99.9] | 99.3 [98.3, >99.9] | >99.9 [99.8, >99.9] | >99.9 [>99.9, >99.9] |
| 19 | >99.9 [>99.9, >99.9] | >99.9 [>99.9, >99.9] | >99.9 [>99.9, >99.9] | >99.9 [>99.9, >99.9] | 99.4 [98.5, >99.9] | >99.9 [99.9, >99.9] | >99.9 [>99.9, >99.9] |
| 20 | >99.9 [>99.9, >99.9] | >99.9 [>99.9, >99.9] | >99.9 [>99.9, >99.9] | >99.9 [>99.9, >99.9] | 99.5 [98.6, >99.9] | >99.9 [>99.9, >99.9] | >99.9 [>99.9, >99.9] |
| 21 | >99.9 [>99.9, >99.9] | >99.9 [>99.9, >99.9] | >99.9 [>99.9, >99.9] | >99.9 [>99.9, >99.9] | 99.6 [98.7, >99.9] | >99.9 [>99.9, >99.9] | >99.9 [>99.9, >99.9] |
| 22 | >99.9 [>99.9, >99.9] | >99.9 [>99.9, >99.9] | >99.9 [>99.9, >99.9] | >99.9 [>99.9, >99.9] | 99.6 [98.8, >99.9] | >99.9 [>99.9, >99.9] | >99.9 [>99.9, >99.9] |
| 23 | >99.9 [>99.9, >99.9] | >99.9 [>99.9, >99.9] | >99.9 [>99.9, >99.9] | >99.9 [>99.9, >99.9] | 99.7 [99.1, >99.9] | >99.9 [>99.9, >99.9] | >99.9 [>99.9, >99.9] |
| 24 | >99.9 [>99.9, >99.9] | >99.9 [>99.9, >99.9] | >99.9 [>99.9, >99.9] | >99.9 [>99.9, >99.9] | 99.9 [99.5, >99.9] | >99.9 [>99.9, >99.9] | >99.9 [>99.9, >99.9] |

*Note. Smoothed cumulative percentiles (SCAM). 95% CI = bootstrap (B = 1,000).*

*Supplementary Table S15. ANX norms - Women by age group.*

| Score | 16-24 [95% CI] | 25-34 [95% CI] | 35-44 [95% CI] | 45-54 [95% CI] | 55-64 [95% CI] | 65-74 [95% CI] | 75+ [95% CI] |
| --- | --- | --- | --- | --- | --- | --- | --- |
| 0 | 50.9 [42.1, 59.9] | 56.7 [48.6, 63.9] | 63.7 [57.7, 70.0] | 60.8 [54.3, 67.4] | 56.0 [50.1, 62.3] | 61.6 [54.7, 68.2] | 51.7 [43.6, 59.4] |
| 1 | 66.6 [58.7, 73.7] | 72.0 [65.6, 78.1] | 76.8 [71.3, 81.8] | 77.1 [72.0, 81.9] | 73.8 [68.8, 78.3] | 74.0 [67.9, 79.6] | 70.1 [62.9, 76.8] |
| 2 | 76.7 [69.2, 83.1] | 82.0 [76.4, 87.6] | 83.6 [78.4, 88.2] | 85.9 [81.2, 90.2] | 82.2 [77.4, 86.7] | 82.4 [77.4, 87.2] | 80.4 [73.6, 86.7] |
| 3 | 83.0 [76.4, 88.4] | 88.0 [83.3, 92.4] | 86.6 [82.1, 90.7] | 90.0 [86.1, 93.5] | 85.5 [81.0, 89.6] | 87.9 [83.8, 91.8] | 85.5 [79.4, 91.1] |
| 4 | 87.1 [81.0, 92.2] | 91.4 [87.2, 95.1] | 88.5 [84.4, 92.2] | 92.3 [88.7, 95.4] | 87.5 [83.7, 91.2] | 91.6 [87.7, 95.1] | 88.7 [83.0, 93.7] |
| 5 | 89.9 [84.2, 94.6] | 93.3 [89.6, 96.8] | 90.4 [86.5, 93.7] | 94.0 [90.6, 96.8] | 89.9 [86.5, 93.2] | 94.1 [90.7, 97.1] | 91.3 [86.6, 95.5] |
| 6 | 92.0 [86.9, 96.0] | 94.2 [90.8, 97.6] | 92.2 [88.6, 95.2] | 95.3 [92.3, 97.8] | 92.4 [89.3, 95.1] | 95.8 [93.0, 98.1] | 93.5 [89.5, 96.9] |
| 7 | 93.7 [89.3, 97.3] | 94.9 [92.0, 98.1] | 93.7 [90.4, 96.6] | 96.3 [93.3, 98.5] | 94.6 [91.7, 96.9] | 97.0 [94.6, 98.8] | 95.3 [91.8, 98.1] |
| 8 | 95.2 [91.7, 98.3] | 95.9 [93.5, 98.5] | 95.0 [92.3, 97.6] | 97.0 [94.3, 99.0] | 96.4 [94.0, 98.3] | 98.0 [96.0, 99.3] | 96.9 [93.9, 99.0] |
| 9 | 96.7 [93.9, 98.9] | 97.1 [94.8, 99.0] | 96.1 [93.7, 98.3] | 97.6 [95.2, 99.4] | 97.7 [96.0, 99.1] | 98.8 [97.4, 99.7] | 98.1 [95.8, 99.6] |
| 10 | 97.8 [95.6, 99.4] | 98.1 [95.9, 99.5] | 96.9 [94.9, 98.8] | 98.1 [96.1, 99.6] | 98.7 [97.4, 99.5] | 99.4 [98.5, 99.9] | 98.9 [97.1, 99.8] |
| 11 | 98.6 [96.7, 99.8] | 98.8 [96.8, 99.8] | 97.5 [95.5, 99.2] | 98.5 [96.8, 99.7] | 99.2 [98.2, 99.8] | 99.8 [99.0, 99.9] | 99.5 [97.9, 99.9] |
| 12 | 99.1 [97.3, >99.9] | 99.1 [97.4, >99.9] | 97.8 [96.0, 99.5] | 98.9 [97.4, 99.8] | 99.6 [98.7, >99.9] | >99.9 [99.4, >99.9] | 99.8 [98.6, >99.9] |
| 13 | 99.4 [97.7, >99.9] | 99.3 [97.9, >99.9] | 98.1 [96.3, 99.6] | 99.2 [98.0, 99.9] | 99.7 [99.0, >99.9] | >99.9 [99.6, >99.9] | 99.9 [99.2, >99.9] |
| 14 | 99.5 [98.0, >99.9] | 99.4 [98.3, >99.9] | 98.4 [96.7, 99.7] | 99.5 [98.5, >99.9] | 99.8 [99.2, >99.9] | >99.9 [99.8, >99.9] | 99.9 [99.5, >99.9] |
| 15 | 99.6 [98.5, >99.9] | 99.6 [98.7, >99.9] | 98.6 [97.0, 99.8] | 99.7 [98.9, >99.9] | 99.9 [99.5, >99.9] | >99.9 [99.9, >99.9] | >99.9 [99.7, >99.9] |
| 16 | 99.7 [99.0, >99.9] | 99.7 [99.1, >99.9] | 98.9 [97.5, 99.9] | 99.8 [99.3, >99.9] | 99.9 [99.7, >99.9] | >99.9 [99.9, >99.9] | >99.9 [99.8, >99.9] |
| 17 | 99.8 [99.3, >99.9] | 99.8 [99.3, >99.9] | 99.1 [97.9, 99.9] | 99.9 [99.6, >99.9] | 99.9 [99.8, >99.9] | >99.9 [>99.9, >99.9] | >99.9 [99.9, >99.9] |
| 18 | 99.9 [99.6, >99.9] | 99.9 [99.6, >99.9] | 99.4 [98.4, >99.9] | 99.9 [99.8, >99.9] | >99.9 [99.9, >99.9] | >99.9 [>99.9, >99.9] | >99.9 [>99.9, >99.9] |
| 19 | 99.9 [99.8, >99.9] | >99.9 [99.8, >99.9] | 99.6 [98.9, >99.9] | >99.9 [99.9, >99.9] | >99.9 [>99.9, >99.9] | >99.9 [>99.9, >99.9] | >99.9 [>99.9, >99.9] |
| 20 | >99.9 [99.9, >99.9] | >99.9 [99.9, >99.9] | 99.7 [99.4, >99.9] | >99.9 [>99.9, >99.9] | >99.9 [>99.9, >99.9] | >99.9 [>99.9, >99.9] | >99.9 [>99.9, >99.9] |
| 21 | >99.9 [>99.9, >99.9] | >99.9 [>99.9, >99.9] | 99.9 [99.7, >99.9] | >99.9 [>99.9, >99.9] | >99.9 [>99.9, >99.9] | >99.9 [>99.9, >99.9] | >99.9 [>99.9, >99.9] |
| 22 | >99.9 [>99.9, >99.9] | >99.9 [>99.9, >99.9] | >99.9 [99.9, >99.9] | >99.9 [>99.9, >99.9] | >99.9 [>99.9, >99.9] | >99.9 [>99.9, >99.9] | >99.9 [>99.9, >99.9] |
| 23 | >99.9 [>99.9, >99.9] | >99.9 [>99.9, >99.9] | >99.9 [>99.9, >99.9] | >99.9 [>99.9, >99.9] | >99.9 [>99.9, >99.9] | >99.9 [>99.9, >99.9] | >99.9 [>99.9, >99.9] |
| 24 | >99.9 [>99.9, >99.9] | >99.9 [>99.9, >99.9] | >99.9 [>99.9, >99.9] | >99.9 [>99.9, >99.9] | >99.9 [>99.9, >99.9] | >99.9 [>99.9, >99.9] | >99.9 [>99.9, >99.9] |

*Note. Smoothed cumulative percentiles (SCAM). 95% CI = bootstrap (B = 1,000).*

*Supplementary Table S16. Cross-cohort comparison: REP 17 (~2009) vs. REP 31 (2020/21).*

| Nr | Item | M 2009 | SD | Skew | Kurt | rit | M 2020/21 | SD | Skew | Kurt | rit | p | delta | 95% CI |
| --- | --- | --- | --- | --- | --- | --- | --- | --- | --- | --- | --- | --- | --- | --- |
| 1 | Faintness/dizziness | 0.19 | 0.51 | 3.2 | 11.43 | 0.53 | 0.15 | 0.48 | 3.78 | 16.66 | 0.61 | 0.019 | -0.02 | [-0.04, -0.01] |
| 2 | No interest in things | 0.36 | 0.69 | 2.37 | 6.42 | 0.69 | 0.38 | 0.73 | 2.28 | 5.53 | 0.72 | 0.264 | 0.01 | [-0.02, 0.03] |
| 3 | Nervousness/shakiness | 0.27 | 0.62 | 2.69 | 8.22 | 0.7 | 0.25 | 0.6 | 2.93 | 9.75 | 0.73 | 0.147 | -0.02 | [-0.04, 0] |
| 4 | Pains in heart/chest | 0.23 | 0.57 | 2.75 | 8.06 | 0.52 | 0.16 | 0.49 | 3.55 | 14.34 | 0.62 | < 0.001 | -0.05 | [-0.07, -0.03] |
| 5 | Feeling lonely | 0.42 | 0.83 | 2.31 | 5.36 | 0.62 | 0.45 | 0.87 | 2.14 | 4.22 | 0.66 | 0.148 | 0.01 | [-0.01, 0.04] |
| 6 | Feeling tense/keyed up | 0.5 | 0.77 | 1.7 | 2.95 | 0.61 | 0.41 | 0.73 | 1.97 | 3.93 | 0.61 | < 0.001 | -0.07 | [-0.09, -0.04] |
| 7 | Nausea/upset stomach | 0.28 | 0.63 | 2.52 | 6.84 | 0.52 | 0.23 | 0.59 | 3.1 | 11.25 | 0.52 | 0.003 | -0.03 | [-0.06, -0.01] |
| 8 | Feeling blue | 0.27 | 0.67 | 2.97 | 9.5 | 0.77 | 0.29 | 0.69 | 2.82 | 8.41 | 0.74 | 0.294 | 0.01 | [-0.01, 0.04] |
| 9 | Suddenly scared | 0.17 | 0.5 | 3.53 | 14.09 | 0.63 | 0.11 | 0.43 | 4.6 | 24.61 | 0.65 | < 0.001 | -0.04 | [-0.06, -0.02] |
| 10 | Trouble getting breath | 0.22 | 0.58 | 3.12 | 11.03 | 0.53 | 0.17 | 0.55 | 3.85 | 16.08 | 0.6 | 0.002 | -0.05 | [-0.06, -0.03] |
| 11 | Feelings of worthlessness | 0.26 | 0.69 | 3.05 | 9.62 | 0.68 | 0.25 | 0.68 | 3.24 | 10.97 | 0.73 | 0.364 | -0.02 | [-0.04, 0] |
| 12 | Spells of terror/panic | 0.1 | 0.39 | 4.69 | 24.83 | 0.67 | 0.1 | 0.41 | 4.96 | 28.7 | 0.68 | 0.61 | 0.00 | [-0.01, 0.02] |
| 13 | Numbness/tingling | 0.22 | 0.6 | 3.28 | 12.2 | 0.52 | 0.2 | 0.58 | 3.62 | 14.72 | 0.59 | 0.184 | -0.02 | [-0.04, 0] |
| 14 | Feeling hopeless | 0.39 | 0.83 | 2.37 | 5.24 | 0.68 | 0.46 | 0.88 | 2.15 | 4.28 | 0.69 | 0.006 | 0.04 | [0.02, 0.07] |
| 15 | Feeling restless | 0.22 | 0.6 | 3.2 | 11.42 | 0.64 | 0.22 | 0.62 | 3.46 | 13.31 | 0.65 | 0.834 | -0.01 | [-0.03, 0.01] |
| 16 | Feeling weak | 0.34 | 0.69 | 2.38 | 6.03 | 0.62 | 0.29 | 0.69 | 2.81 | 8.21 | 0.69 | 0.012 | -0.05 | [-0.07, -0.02] |
| 17 | Thoughts of ending life | 0.07 | 0.34 | 6.04 | 42.79 | 0.57 | 0.06 | 0.34 | 6.87 | 54.58 | 0.53 | 0.425 | -0.01 | [-0.02, 0] |
| 18 | Feeling fearful | 0.18 | 0.53 | 3.42 | 13.23 | 0.63 | 0.16 | 0.49 | 3.7 | 15.3 | 0.68 | 0.067 | -0.02 | [-0.04, 0] |

*Note. Positive = higher in 2020/21. Alpha GSI: REP 17=0.923, REP 31=0.932.*

*Supplementary Table S17. Average variance extracted, Fornell-Larcker criterion, and heterotrait-monotrait ratios for the correlated three-factor model (M2)*

*Panel A. Average variance extracted*

| Factor | AVE | sqrt(AVE) |
| --- | --- | --- |
| SOM | .685 | .828 |
| DEP | .750 | .866 |
| ANX | .734 | .857 |

*Panel B. Fornell-Larcker criterion*

| Factor pair | Latent r | sqrt(AVE) Factor 1 | sqrt(AVE) Factor 2 | Criterion met |
| --- | --- | --- | --- | --- |
| SOM–DEP | .824 | .828 | .866 | Yes |
| SOM–ANX | .895 | .828 | .857 | No |
| DEP–ANX | .913 | .866 | .857 | No |

*Panel C. Heterotrait-monotrait ratios*

| Factor pair | HTMT |
| --- | --- |
| SOM–DEP | .832 |
| SOM–ANX | .897 |
| DEP–ANX | .919 |

*Note. N = 2,519. AVE = average variance extracted (mean of squared standardized loadings under the correlated three-factor model, WLSMV estimation). The Fornell-Larcker criterion is met when the square root of the AVE of both factors exceeds their latent correlation. HTMT = heterotrait-monotrait ratio of correlations; conventional cutoffs are .85 (strict) and .90 [liberal; (57)]. SOM = somatization; DEP = depression; ANX = anxiety. The heterotrait-monotrait ratio for depression-anxiety (.919) exceeded both cutoffs, indicating limited discriminant validity between these two dimensions at the latent level.*

## Supplementary Figures

*Supplementary Figure 1. MGCFA models for measurement invariance Analysis*

*Note.*

*(1)	Configural invariance: Same CFA model fit in all groups;*

*(2)	Threshold Invariance: thresholds τ1i…τ4i constrained to be equal across groups;*

*(3)	Weak Invariance: additionally, factor loadings λi constrained to be equal across groups;*

*(4)	Strong Invariance: additionally, intercepts constrained to be equal across groups;*

*(5)	Full Invariance: additionally, residual variances ϵi constrained to be equal across groups.*

*Supplementary Figure 2. Path diagrams of the unidimensional model (M1), correlated three-factor model (M2), and higher-order model (M3) of the BSI-18.*
